# The WFS1‐ZnT3‐Zn^2+^ Axis Regulates the Vicious Cycle of Obesity and Depression

**DOI:** 10.1002/advs.202403405

**Published:** 2024-09-11

**Authors:** Mengting Gong, Yulin Fang, Kaijiang Yang, Fei Yuan, Rui Hu, Yajuan Su, Yiling Yang, Wenjun Xu, Qing Ma, Jiaxue Cha, Ru Zhang, Zhen‐Ning Zhang, Weida Li

**Affiliations:** ^1^ Institute for Regenerative Medicine State Key Laboratory of Cardiology and Medical Innovation Center Shanghai East Hospital Frontier Science Center for Stem Cell Research Shanghai Key Laboratory of Signaling and Disease Research School of Life Sciences and Technology Tongji University Shanghai 200092 China; ^2^ Shanghai Key Laboratory of Signaling and Disease Research School of Life Sciences and Technology Tongji University Shanghai 200092 China

**Keywords:** cerebral organoid, depression, obesity, WFS1, ZnT3

## Abstract

Obesity, a growing global health concern, is closely linked to depression. However, the neural mechanism of association between obesity and depression remains poorly understood. In this study, neural‐specific *WFS1* deficiency exacerbates the vicious cycle of obesity and depression in mice fed a high‐fat diet (HFD), positioning *WFS1* as a crucial factor in this cycle. Through human pluripotent stem cells (hESCs) neural differentiation, it is demonstrated that *WFS1* regulates Zn^2+^ homeostasis and the apoptosis of neural progenitor cells (NPCs) and cerebral organoids by inhibiting the zinc transporter *ZnT3* under the situation of dysregulated lipid metabolism. Notably, riluzole regulates *ZnT3* expression to maintain zinc homeostasis and protect NPCs from lipotoxicity‐induced cell death. Importantly, riluzole, a therapeutic molecule targeting the nervous system, in vivo administration prevents HFD‐induced obesity and associated depression. Thus, a WFS1‐ZnT3‐Zn^2+^ axis critical is demonstrated for the vicious cycle of obesity and depression and that riluzole may have the potential to reverse this process against obesity and depression.

## Graphical abstract

Obesity and depression are attracting worldwide attention, but mechanisms of this vicious cycle remain unclear. The stdy shows that neural‐specific *WFS1* deletion exacerbates high‐fat diet‐induced obesity and depression via modulating zinc homeostasis. By contrary, riluzole releases this vicious cycle. Mechanically, *WFS1* deficiency exacerbates lipotoxicity‐induced apoptosis in human cerebral organoids and 2D neural progenitor cells via the WFS1‐ATF4‐ZnT3 signaling axis.

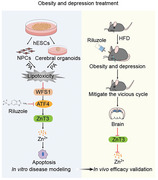

## 1 Introduction

Obesity and depression are two significant contributors to diseases globally.^[^ ^1^ ^]^ A meta‐analysis of 19 studies reveals a vicious cycle of depression and obesity, with a higher risk of being obese in depressed individuals and an increased risk of being depressed in obese individuals.^[^ ^2^ ^]^ In Australia, the prevalence of depression among obese patients reaches 23%.^[^ ^3^ ^]^ Studies have shown that men with both depression and obesity have a 68% excess risk of diabetes and a 57% excess risk of hypertension compared to the sum of independent risks.^[^ ^4^ ^]^ Their vicious cycle often results in the co‐occurrence of depression and obesity, and then intensifies the risk of disease.^[^ ^4^, ^5^, ^6^ ^]^

Obesity is correlated with cognitive decline,^[^ ^7^ ^]^ mainly reflected in structural brain abnormalities including lower brain volume,^[^ ^8^, ^9^, ^10^, ^11^ ^]^ brain atrophy,^[^ ^12^ ^]^ and white matter hyperintense.^[^ ^13^ ^]^ One study in the systematic review, which assesses depression by PHQ‐9, estimates that obese adults have a 2.5 times higher risk of depression.^[^ ^6^ ^]^ A high‐fat diet is a major risk factor that induces obesity and type 2 diabetes (T2D).^[^ ^14^, ^15^ ^]^ These current studies evaluated that prolonged HFD intake increases the risk of anxiety behavior and depression‐like behavior in mice.^[^ ^16^, ^17^, ^18^ ^]^ Despite previous studies have suggested that obesity can affect cognitive function, particularly depression, the underlying mechanisms are unclear. Mental deficiency is a common behavior characteristic in obese patients and animal models. Studies have shown that some neural vulnerability factors may increase the risk of overeating and obesity.^[^ ^19^, ^20^ ^]^ *WFS1* is highly expressed in pancreatic islets and brain.^[^ ^21^, ^22^ ^]^ *WFS1* variants are linked to metabolic traits and T2D.^[^ ^23^ ^]^ Previous results reveal that *WFS1* variants (rs1046322) are significantly associated with BMI and waist circumference (WC) in the Southeast Asian population.^[^ ^24^ ^]^ Moreover, the Wfs1 mutation increases the susceptibility to HFD‐induced diabetes or metabolic syndrome in mice.^[^ ^25^ ^]^ Despite this, there is a lack of direct evidence regarding the associations between *WFS1* and obesity. Additionally, our previous research reveals that *Wfs1* loss in the nervous system causes depression‐like behavior in mice.^[^ ^26^ ^]^ Studies in other species have demonstrated that knocking down *Wfs1* leads to age‐dependent behavioral deficits and neurodegeneration in the fly brain.^[^ ^27^ ^]^ Previous reports have also found a significant reduction of the brain stem in *Wfs1* ^−/−^ mice and rats.^[^ ^28^, ^29^ ^]^ Consequently, these reports across multiple species indicate that *WFS1* plays a conserved and essential role in brain development and neurodegeneration. However, whether *WFS1* deficiency regulates obesity remains unclear in the nervous system.

Intracellular Zn^2+^ increase is associated with neuronal cell death and brain injuries.^[^ ^30^ ^]^ Previous studies have found that the increase of intracellular Zn^2+^ concentration (≥0.1 × 10^−6^ m) occurs in cerebral ischemia and brain injuries.^[^ ^31^ ^]^ Moreover, excessive Zn^2+^ accumulation has been linked to excessive lipid accumulation in the liver, leading to lipotoxicity.^[^ ^32^, ^33^ ^]^ These findings suggest that excessive zinc may impair neural cells and contribute to lipotoxicity, encouraging us to investigate the correlation between excessive zinc in the brain and obesity.

Here, we utilize 3D human cerebral organoids and 2D neural progenitor cells derived from human embryonic stem cells harboring *WFS1* deficiency and *Wfs1* conditional knockout mice (*Wfs1* ^flox/flox^ X Nestin‐Cre, CKO) exposed to HFD to examine the vicious cycle of obesity and depression. Our results reveal that neural‐specific *WFS1* deletion exacerbates HFD‐induced obesity and depression via modulating zinc homeostasis. Mechanically, *WFS1* deficiency exacerbates lipotoxicity‐induced apoptosis in 3D human cerebral organoids and 2D NPCs via the WFS1‐ATF4‐ZnT3 signaling axis regulating zinc homeostasis. Importantly, riluzole, which targets the nervous system, down‐regulates zinc transportation by inhibiting *ZnT3* expression and rescues the apoptosis in 3D human cerebral organoids and 2D NPCs induced by *WFS1* deficiency. Furthermore, riluzole not only attenuates HFD‐induced depressive‐like behaviors but also alleviates obesity. Thus, our results reveal mechanistic insight into the vicious cycle of obesity and depression induced by neural‐specific *WFS1* deficiency and propose a therapeutic approach to counteract obesity and depression with riluzole.

## 2 Results

### 2.1 Neural *WFS1* Deficiency Exacerbates the Vicious Cycle of Obesity and Depression

**Figure 1:**
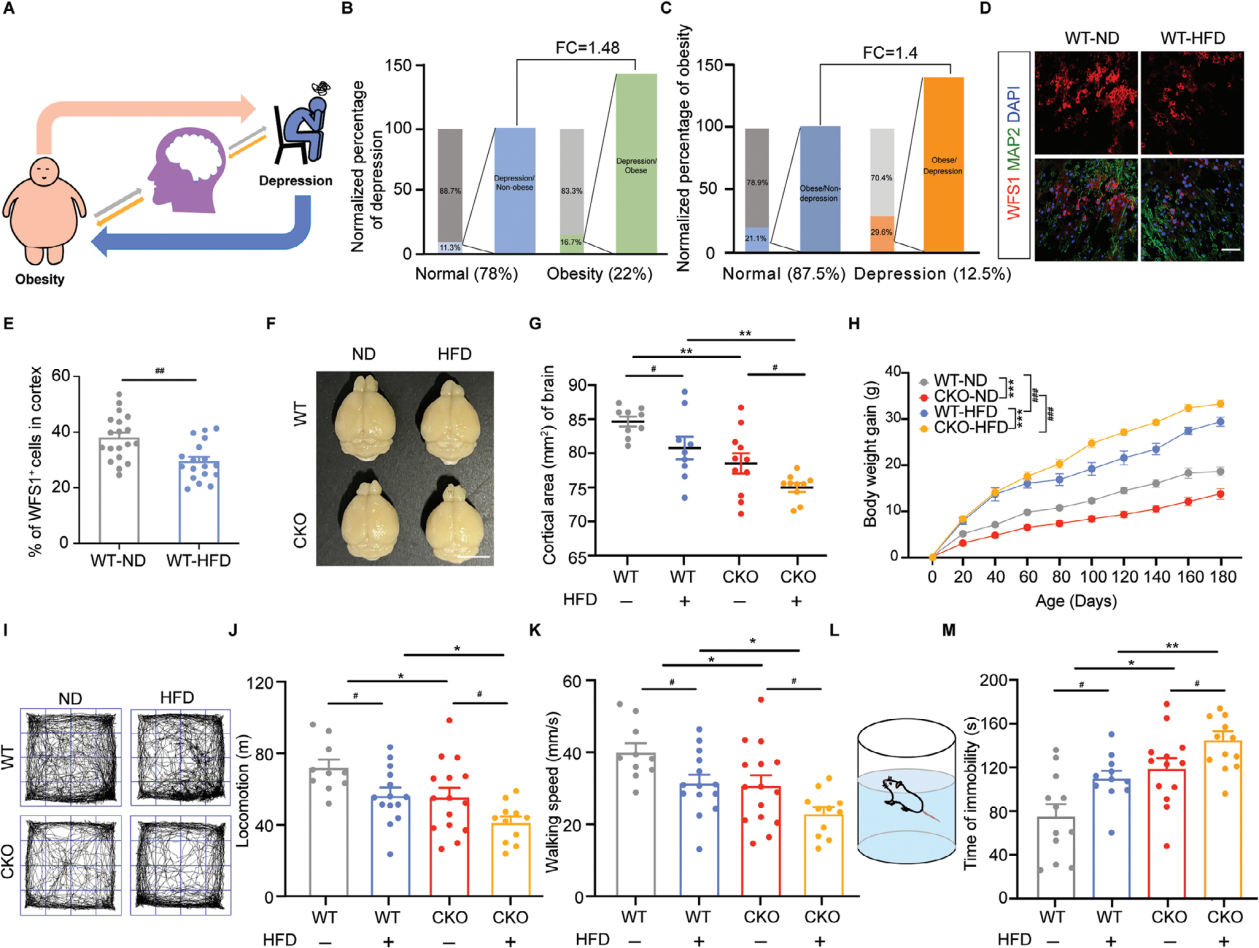
Neural‐specific deficiency of *WFS1* exacerbates the bidirectional relationship between obesity and depression. A) Schematic diagram of the vicious cycle of obesity and depression. B) Quantification of the proportion of depression in normal and obese populations. C) Quantification of the proportion of obesity in normal and depressed populations. D) Immunostaining for WFS1 (red), MAP2 (green), and DAPI (blue) on the cerebral cortex in ND or HFD mice. Scale bar, 50 µm. E) Quantification of the percentage of WFS1^+^ cells on the cerebral cortex in ND or HFD‐fed WT mice (WT + ND, *n* = 19; WT + HFD, *n* = 18). F) Representative bright‐field images of the brains of ND or HFD‐fed WT or CKO mice. Scale bar, 5 mm. G) Quantification of the cortical area (mm^2^) of the brains of ND or HFD‐fed WT or CKO mice (WT + ND, *n* = 9; WT + HFD, *n* = 9; CKO + ND, *n* = 11; CKO + HFD, *n* = 10). H) Body weight gain in ND or HFD‐fed WT or CKO mice from day 0 to day 180 (WT + ND, *n* = 8; WT + HFD, *n* = 7; CKO + ND, *n* = 10; CKO + HFD, *n* = 13). I) Representative movement traces of ND or HFD‐fed WT or CKO mice in the open field test. Analysis of J) the locomotion and K) the walking speed during open field test in ND or HFD‐fed WT or CKO mice (WT + ND, *n* = 10; WT + HFD, *n* = 14; CKO + ND, *n* = 15; CKO + HFD, *n* = 11). L) Schematic of the forced swimming test. M) Analysis of the immobility time during the forced swimming test in ND or HFD‐fed WT or CKO mice (WT + ND, *n* = 12; WT + HFD, *n* = 11; CKO + ND, *n* = 13; CKO + HFD, *n* = 12). Data were presented as mean ± SEM. Significance was calculated by E,G,J,K,M) unpaired two‐tailed Student's *t*‐test, and H) two‐way ANOVA. ^*^ *p* < 0.05, ^**^ *p* < 0.01, and ^***^ *p* < 0.001 were considered to be significant for the comparison of WT and CKO; ^#^ *p* < 0.05, ^##^ *p* < 0.01, and ^###^ *p* < 0.001 were considered to be significant for comparison of ND and HFD.

Obesity and depression are among the leading causes of illness that seriously affect people's lives across the globe.^[^ ^1^ ^]^ We analyzed the prevalence of obesity and depression among Australian men, building upon earlier findings that reported,^[^ ^4^ ^]^ that the risk of depression in obese individuals was 1.48 times that of the general population. Similarly, depressed individuals were 1.4 times more likely to be obese than those without depression (**Figure** **1**A–C). These results indicated that a bidirectional relationship often results in comorbid depression and obesity, which is a vicious cycle. Reports from multiple species indicate that *WFS1* has a conserved and essential role in brain development and mental health.^[^ ^26^, ^28^, ^34^ ^]^ Previous results demonstrate that the *WFS1* variant (rs1046322) is significantly associated with BMI and WC in the Southeast Asian population.^[^ ^24^ ^]^ We found a significant reduction of WFS1 positive cells in the cortex of HFD mice (Figure 1D,E). To explore the impact of *WFS1* deficiency in the nervous system on obesity, *Wfs1*‐flox mice (*Wfs1^f/f^)* were crossed with *Nestin‐Cre* transgenic mice to generate the conditional *Wfs1* knockout (CKO) mice. *Wfs1* deletion in the nervous system in CKO mice was confirmed by immunostaining (Figure S1A,B, Supporting Information). Notably, *WFS1* knockout showed a reduction in brain size in mice and high‐fat feeding exacerbated this reduction in mice (Figure 1F,G). To elaborate on the vicious cycle of obesity and depression, the obesity model was established by feeding HFD, and depression‐like behavioral tests were conducted including open field tests, forced swimming tests, and so on. The body weight gain of CKO‐normal diet (ND) mice from 6 to 30 weeks was much lower than that of WT‐ND, but the body weight gain of CKO‐HFD mice was higher than WT‐HFD suggesting that *Wfs1* deficiency increased the susceptibility to HFD‐inducing obesity (Figure 1H). Our previous research revealed that *Wfs1* loss in the nervous system causes depression‐like behavior in mice with ND.^[^ ^26^ ^]^ In the open field test, WT‐HFD mice showed a significant decrease in locomotion and walking speeds than WT‐ND mice, which was more pronounced in CKO‐HFD mice (Figure 1I–K). In the forced swimming test, WT‐HFD mice with longer immobility duration showed more serious depression‐like behavior, which was accentuated in CKO‐HFD, indicating that mice developed depressive‐like behavior induced by HFD and exacerbated by *Wfs1* deficiency (Figure 1L,M). Collectively, these observations indicate that HFD‐induced obesity and depressive‐like behavior are exacerbated by *Wfs1* deficiency.

### 2.2 *WFS1* Deficiency in Human Cerebral Organoids Exacerbates Lipotoxicity‐Induced Neural Apoptosis by Regulating Zinc Homeostasis

**Figure 2:**
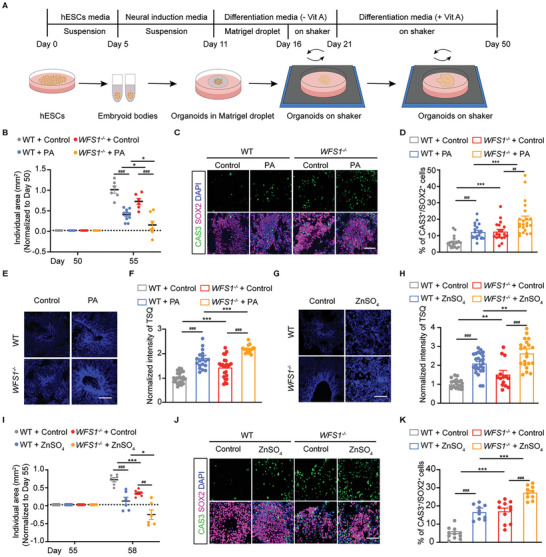
*WFS1* deficiency exacerbates lipotoxicity‐induced apoptosis of NPCs in human organoids by regulating zinc homeostasis. A) Schematic of the differentiation of cerebral organoids. B) Quantification of the increased individual area (mm^2^) of WT and *WFS1* ^−/−^ cerebral organoids treated with vehicle or 1 × 10^−3^ m PA from day 50 to day 55 (WT, *n* = 6; WT + PA, *n* = 10; *WFS1* ^−/−^, *n* = 6; *WFS1* ^−/−^ + PA, *n* = 7). C) Immunostaining for CAS3 (green), SOX2 (magenta), and DAPI (blue) in WT and *WFS1* ^−/−^ cerebral organoids treated with vehicle or 1 × 10^−3^ m PA. Scale bar, 50 µm. D) Quantification of the percentage of CAS3^+^ cells among the total number of SOX2 ^+^ cells in WT and *WFS1* ^−/−^ cerebral organoids treated with vehicle or 1 × 10^−3^ m PA (WT, *n* = 20; WT + PA, *n* = 21; *WFS1* ^−/−^, *n* = 19; *WFS1* ^−/−^ + PA, *n* = 22). E) Representative images of TSQ in WT and *WFS1* ^−/−^ cerebral organoids treated with vehicle or 1 × 10^−3^ m PA from day 50 to day 55. Scale bar 50 µm. F) Quantification of the normalized intensity of TSQ in WT and *WFS1* ^−/−^ cerebral organoids treated with vehicle or 1 × 10^−3^ m PA from day 50 to day 55 (WT, *n* = 25; WT + PA, *n* = 19; *WFS1* ^−/−^, *n* = 24; *WFS1* ^−/−^ + PA, *n* = 17). G) Representative images of TSQ in WT and *WFS1* ^−/−^ cerebral organoids treated with vehicle or 200 × 10^−6^ m ZnSO_4_ from day 55 to day 58. Scale bar 50 µm. H) Quantification of the normalized intensity of TSQ of WT and *WFS1* ^−/−^ cerebral organoids treated with vehicle or 200 × 10^−6^ m ZnSO_4_ from day 55 to day 58 (WT, *n* = 21; WT + ZnSO_4_, *n* = 26; *WFS1* ^−/−^, *n* = 14; *WFS1* ^−/−^ + ZnSO_4_, *n* = 21). I) Quantification of the increased individual area (mm^2^) of WT and *WFS1* ^−/−^ cerebral organoids treated with vehicle or 200 × 10^−6^ m ZnSO_4_ from day 55 to day 58 (*n* = 6). J) Immunostaining for CAS3 (green), SOX2 (magenta), and DAPI (blue) in WT and *WFS1* ^−/−^ cerebral organoids treated with vehicle or 200 × 10^−6^ m ZnSO_4_. Scale bar, 50 µm. K) Quantification of the percentage of CAS3^+^ cells among the total number of SOX2^+^ cells in WT and *WFS1* ^−/−^ cerebral organoids treated with vehicle or 200 × 10^−6^ m ZnSO_4_ (WT, *n* = 11; WT + ZnSO_4_, *n* = 10; *WFS1* ^−/−^, *n* = 11; *WFS1* ^−/−^ + ZnSO_4_, *n* = 10). Data were presented as mean ± SEM. Significance was calculated by unpaired two‐tailed Student's *t*‐test. ^*^ *p* < 0.05, ^**^ *p* < 0.01, and ^***^ *p* < 0.001 were considered to be significant for the comparison of WT and *WFS1* ^−/−^. ^#^ *p* < 0.05, ^##^ *p* < 0.01, and ^###^ *p* < 0.001 were considered to be significant for comparison of PA or ZnSO_4_ treatment.

To recapitulate the pivotal role of *WFS1* in human obesity and depression, we employed a 3D culture system for deriving human cerebral organoids in vitro. Cerebral organoids generated from WT and *WFS1^−/−^* H1 hESCs were used to visually assess the effects of *WFS1* on the nervous system in vitro (**Figure** **2**A). During the growth of cerebral organoids, *WFS1*‐deficient organoids consistently displayed a smaller size compared to WT and exhibited a significant difference at Day 50 (Figure S2A,B, Supporting Information). Palmitic acid (PA) is the most common saturated fatty acid to induce obesity. PA‐induced lipotoxicity has been reported to contribute to neuropathy in T2D^[^ ^35^ ^]^ and neurodegenerative diseases.^[^ ^36^, ^37^ ^]^ To more accurately simulate the pathological environment of obesity in humans to explore the regulation of *WFS1* in obesity, we added 1 × 10^−3^ m PA to the cerebral organoids at 50 d and measured the size of the cerebral organoids 5 d later. *WFS1* deficiency resulted in a slowed growth rate of cerebral organoids and became even slower with the addition of PA (Figure 2B). Studies have shown that PA causes apoptosis in hypothalamic neurons.^[^ ^38^, ^39^ ^]^ Subsequent immunofluorescence staining revealed PA‐induced apoptosis in neural rosettes and *WFS1* deficiency exacerbated apoptosis (Figure 2C,D). To explore how *WFS1* deficiency and lipotoxicity induce apoptosis, we examined zinc levels in cerebral organoids by TSQ staining. The results showed a significant increase in zinc levels in PA‐treated organoids, compared to the control group. PA treatment in *WFS1*‐deficient cerebral organoids led to higher levels of zinc, implying that zinc may play a critical role in cell death regulated by *WFS1* (Figure 2E,F). Therefore, to explore whether increased zinc could directly induce apoptosis, we directly employed 200 × 10^−6^ m exogenous ZnSO_4_ to cerebral organoids. Elevated zinc led to a significant induction of apoptosis and a reduction in cerebral organoid size (Figure 2G–K). In summary, these findings suggest that *WFS1* deficiency exacerbates lipotoxicity‐induced apoptosis of NPCs in human cerebral organoids by regulating zinc homeostasis.

### 2.3 The WFS1‐ATF4‐ZnT3 Signaling Axis Regulates Zinc Homeostasis in NPCs

**Figure 3:**
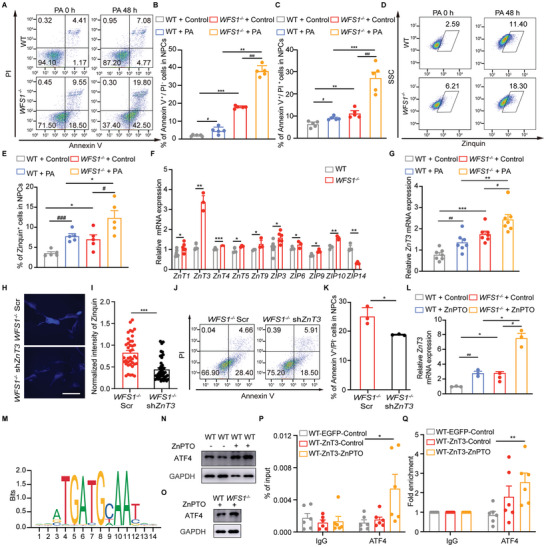
The WFS1‐ATF4‐ZnT3 signaling axis regulates zinc homeostasis in NPCs. A) Representative FACS plots and quantification of death cells (B, Annexin V^+^/PI^−^; C, Annexin V^+^/PI^+^) of WT and *WFS1* ^−/−^ NPCs treated with 200 × 10^−6^ m PA or vehicle for 48 h by double‐staining with Annexin V and PI (*n* = 5). Representative D) FACS plots and E) quantifications of the percentage of Zinquin^+^ cells in WT and *WFS1* ^−/−^ NPCs treated with 200 × 10^−6^ m PA or vehicle for 48 h (*n* = 5). F) Quantitative real‐time PCR analysis of *ZnT* and *ZIP* family in WT and *WFS1* ^−/−^ NPCs (*ZnT1*, *n* = 6; *ZnT3*, *n* = 3; *ZnT4*, *n* = 3; *ZnT5*, *n* = 3; *ZnT9*, *n* = 3; *ZIP 3*, *n* = 6; *ZIP6*, *n* = 3; *ZIP9*, *n* = 3; *ZIP10*, *n* = 3; *ZIP14*, *n* = 3). G) Quantitative real‐time PCR analysis of *ZnT3* in WT and *WFS1* ^−/−^ NPCs treated with 200 × 10^−6^ m PA or vehicle for 48 h (*n* = 7). H) Representative images of Zinquin and I) quantification of the normalized intensity of Zinquin in *WFS1* ^−/−^ NPCs infected with Lenti‐sh*ZnT3* or Scr (*WFS1* ^−/−^ Scr, *n* = 40; *WFS1* ^−/−^ sh*ZnT3*, *n* = 42). Scale bar 50 µm. J) Representative FACS plots and quantifications (K, Annexin V^+^/PI^−^) of *WFS1* ^−/−^ NPCs infected with Lenti‐sh*ZnT3* or Scr by double‐staining with Annexin V and PI (*n* = 3 per group). L) Quantitative real‐time PCR analysis of *ZnT3* in WT and *WFS1* ^−/−^ NPCs treated with 5 × 10^−6^ m ZnPTO or vehicle (*n* = 3). M) *ZnT3* promotor binding motifs of ATF4. N) Representative immunoblots of ATF4 and GAPDH in WT NPCs treated with 5 × 10^−6^ m ZnPTO or vehicle. O) Representative immunoblots of ATF4 and GAPDH in WT and *WFS1* ^−/−^ NPCs treated with 5 × 10^−6^ ZnPTO or vehicle. P) ChIP analysis, and Q) relative fold change of ATF4 enrichment at *ZnT3* promotor in ZnPTO‐treated ZnT3 overexpressed NPCs (*n* = 6). Data were presented as mean ± SEM. Significance was calculated by unpaired two‐tailed Student's *t*‐test. ^*^ *p* < 0.05, ^**^ *p* < 0.01, and ^***^ *p* < 0.001 were considered to be significant for comparison of WT and *WFS1* ^−/−^ or *WFS1* ^−/−^ Scr and *WFS1* ^−/−^ sh*ZnT3* or WT‐EGFP‐control and WT‐ZnT3‐ZnPTO; ^#^ *p* < 0.05, ^##^ *p* < 0.01, and ^###^ *p* < 0.001 were considered to be significant for comparison of PA or ZnPTO treatment.

Due to the large variety of cell types in cerebral organoids, we used 2D NPCs derived from hESCs to investigate the response of zinc in PA‐induced apoptosis in NPCs. The expression of *WFS1* initially increased and then decreased after 24 h and 48 h of PA treatment, suggesting *WFS1* initially plays a stress‐protective role in response to PA in NPCs (Figure S3A–C, Supporting Information). PA aggravated the cell death induced by *WFS1* deficiency, along with the elevation of zinc levels in NPCs, suggesting the effect of *WFS1* regulating PA‐induced apoptosis (**Figure** **3**A–E). Conversely, *WFS1* overexpression rescued the cell death induced by *WFS1* deficiency, along with the decreasing zinc ion levels in NPCs (Figure S3D–H, Supporting Information). Furthermore, we found that excessive zinc caused cell death in NPCs, as evidenced by the addition of zinc pyrithione (ZnPTO), a Zn^2+^ specific ionophore, its effect reversed by mental ion chelator (TPEN) (Figure S4A–E, Supporting Information). To identify the specific zinc transporter that mediated the effect, we detected the expression of zinc transporter family genes in *WFS1^−/−^* NPCs. *ZnT3* was the zinc transporter exhibiting the most significant upregulation in *WFS1^−/−^* NPCs (Figure 3F), and *ZnT3* upregulation was much more obvious with PA after 48 h treatment (Figure 3G). These findings suggested that *WFS1* regulates cellular zinc levels induced by PA through *ZnT3*. To validate this hypothesis, we constructed a *ZnT3* knockdown plasmid and found that the apoptosis was attenuated with reduced zinc level by *ZnT3* knockdown in *WFS1^−/−^* NPCs (Figure 3H–K). To further explore the direct relationship that *WFS1* regulates zinc through *ZnT3*, treatment with 5 × 10^−6^ m ZnPTO for 3 h resulted in a significant increase in *ZnT3* expression, which was particularly pronounced in *WFS1^−/−^* NPCs (Figure 3L). As an endoplasmic reticulum transmembrane protein, *WFS1* is closely related to the unfolded protein response (UPR). Among the major components for sensing stress, JASPAR datasets analysis identified a potential binding site for activating transcription factor 4 (ATF4) in the promoter region of *ZnT3* (Figure 3M). The expression of ATF4, a major molecular component of the UPR signaling pathway, was increased by 5 × 10^−6^ m ZnPTO treatment in WT NPCs, which was more pronounced in *WFS1^−/−^* NPCs (Figure 3N,O). Chromatin immunoprecipitation (ChIP) results revealed the interaction of ATF4 and *ZnT3* promoter that was enhanced by the ZnPTO treatment (Figure 3P,Q), which suggested that *WFS1* through ATF4‐ZnT3 signaling axis regulates zinc homeostasis in NPCs.

### 2.4 Riluzole Reduces NPCs Apoptosis by Regulating Zinc Homeostasis in Human Cerebral Organoids

**Figure 4:**
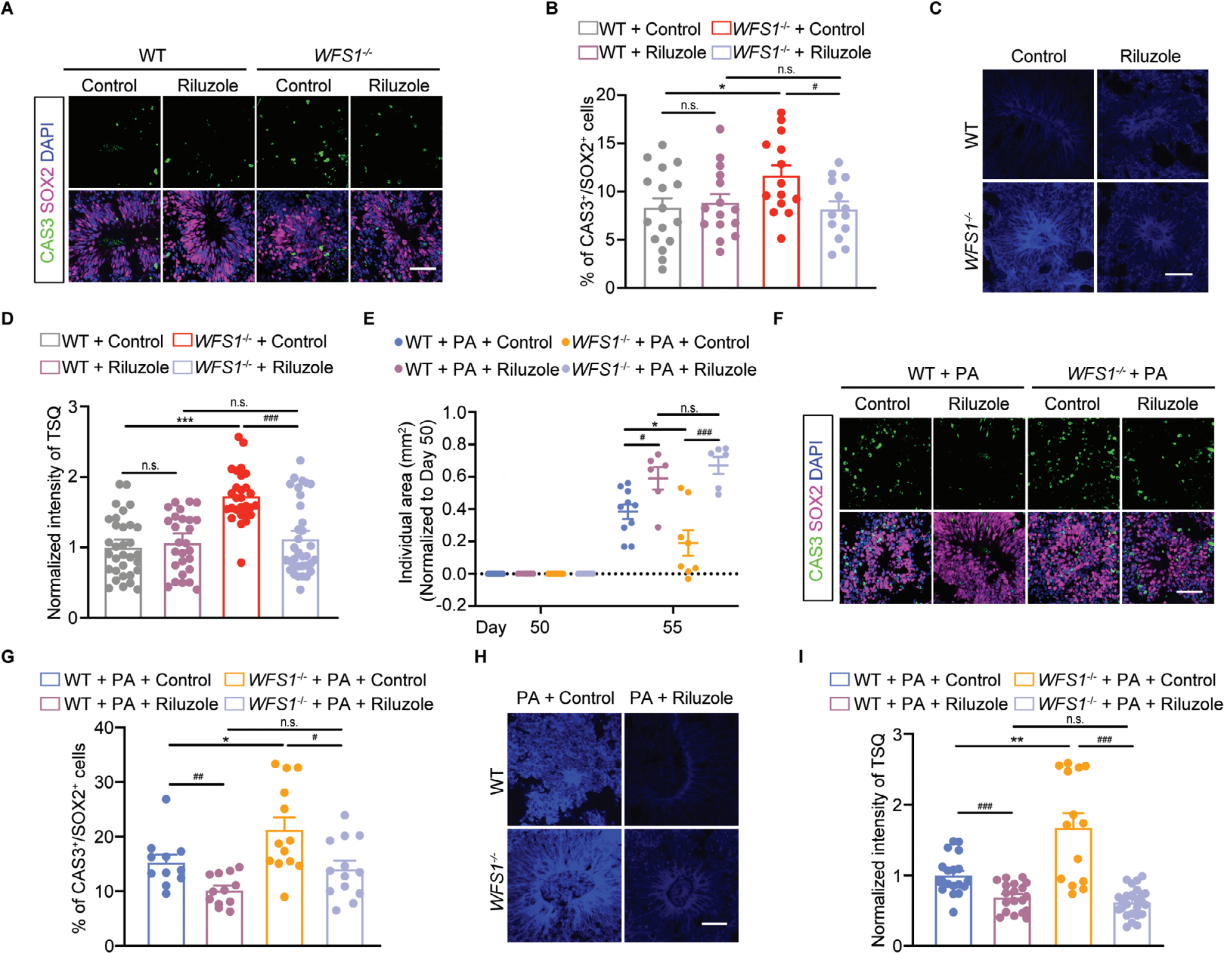
Riluzole reduces NPCs apoptosis by regulating zinc homeostasis in human cerebral organoids. A) Immunostaining for CAS3 (green), SOX2 (magenta), and DAPI (blue) in WT and *WFS1* ^−/−^ cerebral organoids treated with 5 × 10^−6^ riluzole or vehicle. Scale bar, 50 µm. B) Quantification of the percentage of CAS3^+^ cells among the total number of SOX2^+^ cells in WT and *WFS1* ^−/−^ cerebral organoids treated with 5 × 10^−6^ m riluzole or vehicle (WT + control, *n* = 16; WT + riluzole, *n* = 15; *WFS1* ^−/−^ + control, *n* = 14; *WFS1* ^−/−^ + riluzole, *n* = 13). C) Representative images of TSQ fluorescence in WT and *WFS1* ^−/−^ cerebral organoids treated with 5 × 10^−6^ m riluzole or vehicle. Scale bar, 50 µm. D) Quantification of the normalized intensity of TSQ of WT and *WFS1* ^−/−^ cerebral organoids treated with 5 × 10^−6^ m riluzole or vehicle (WT + control, *n* = 32; WT + riluzole, n = 27; *WFS1* ^−/−^ + control, *n* = 28; *WFS1* ^−/−^ + riluzole, *n* = 34). E) Quantification of the increased individual area (mm^2^) of WT and *WFS1* ^−/−^ cerebral organoids treated with 5 × 10^−6^ m riluzole or vehicle from day 50 to day 55 (WT + PA + control, *n* = 10; WT + PA + riluzole, n = 6; *WFS1* ^−/−^ + PA + Control, *n* = 8; *WFS1* ^−/−^ + PA + riluzole, *n* = 6). F) Immunostaining for CAS3 (green), SOX2 (magenta), and DAPI (blue) in 1 × 10^−3^ m PA‐treated WT and *WFS1* ^−/−^ cerebral organoids treated with 5 × 10^−6^ m riluzole or vehicle. Scale bar, 50 µm. G) Quantification of the percentage of CAS3^+^ cells among the total number of SOX2^+^ cells in 1 × 10^−3^ m PA‐treated WT and *WFS1* ^−/−^ cerebral organoids treated with 5 × 10^−6^ m riluzole or vehicle (WT + PA + control, *n* = 11; WT + PA + riluzole, n = 12; *WFS1* ^−/−^ + PA + control, *n* = 13; *WFS1* ^−/−^ + PA + riluzole, *n* = 13). H) Representative images of TSQ fluorescence in 1 × 10^−3^ m PA‐treated WT and *WFS1* ^−/−^ cerebral organoids treated with 5 × 10^−6^ m riluzole or vehicle. Scale bar 50 µm. I) Quantification of the normalized intensity of TSQ of 1 × 10^−3^ m PA‐treated WT and *WFS1* ^−/−^ cerebral organoids treated with 5 × 10^−6^ m riluzole or vehicle (WT + PA + Control, *n* = 19; WT + PA + riluzole, *n* = 19; *WFS1* ^−/−^ + PA + control, *n* = 14; *WFS1* ^−/−^ + PA + riluzole, *n* = 25). Data were presented as mean ± SEM. Significance was calculated by unpaired two‐tailed Student's *t*‐test. ^*^ *p* < 0.05, ^**^ *p* < 0.01, and ^***^ *p* < 0.001 were considered to be significant for comparison of WT and *WFS1* ^−/−^; ^#^ *p* < 0.05, ^##^ *p* < 0.01, and ^###^ *p* < 0.001 were considered to be significant for comparison of riluzole treatment.

Riluzole, a glutamate modulator is approved for amyotrophic lateral sclerosis (ALS).^[^ ^40^, ^41^ ^]^ Our previous study showed that the disrupted synapse formation and function in *WFS1‐*deficient human cerebral organoids could be efficiently reversed with riluzole treatment.^[^ ^26^ ^]^ To verify the impact of riluzole on apoptosis in the cerebral organoids induced by *WFS1* deficiency, human cerebral organoids were treated with 5 × 10^−6^ m riluzole for 5 d. Immunofluorescence staining results showed the proportion of CAS3^+^ in SOX2^+^ cells decreased significantly after the treatment of *WFS1* ^−/−^ cerebral organoids with riluzole, suggesting that riluzole rescues apoptosis induced by *WFS1* deficiency (**Figure** **4**A,B). Meanwhile, TSQ staining of cerebral organoids demonstrated that the upregulation of zinc levels due to *WFS1* deficiency was inhibited by riluzole (Figure 4C,D).

Given that in previous experiments, lipotoxicity exacerbated apoptosis in *WFS1* ^−/−^ cerebral organoids and led to a decrease in size, we further examined whether riluzole could mitigate the effect of lipotoxicity. riluzole treatment rescued cerebral organoid size and apoptosis of NPCs by reducing zinc levels from lipotoxicity in *WFS1* ^−/−^ human cerebral organoids (Figure 4E–I). Our previous study showed the decreased size of *WFS1*‐deficient organoids since Day 60 recapitulates the reduced brain volume observed in WS patients.^[^ ^26^ ^]^ Meanwhile, disrupted synapse formation in *WFS1*‐deficient organoids underlies WS psychiatric disorders,^[^ ^26^ ^]^ and patients with WS have been reported to suffer from a variety of psychiatric disorders including depression.^[^ ^42^ ^]^ Further, we performed the immunostaining for the presynaptic marker Synapsin 1 (SYN1), and the postsynaptic marker PSD95 and found that the percentage of SYN1/PSD95 colocalized puncta to SYN1 puncta was significantly decreased in *WFS1*‐deficient organoids as compared to WT organoids (Figure S5A,B, Supporting Information). Besides, brain‐derived neurotrophic factor (BDNF) plays a pivotal role inside the brain, such as neuronal activity and enhancing both synaptic and structural plasticity.^[^ ^43^, ^44^ ^]^ We found that the population of BDNF^+^ neurons was significantly decreased in *WFS1‐*deficient organoids as compared to WT organoids, which was rescued by riluzole (Figure S5C,D, Supporting Information). BDNF may regulate functional connectivity and synaptic plasticity in the brain, leading to antidepressant‐like effects. These results suggest the relevance of the phenotypic changes in organoids to depression.

Next, we examined the effect of riluzole on lipotoxicity‐induced apoptosis in 2D differentiated NPCs. Riluzole inhibited *ATF4* and *ZnT3* expression, restored zinc homeostasis and suppressed apoptosis that were exacerbated by PA in WT and *WFS1^−/−^* NPCs (Figure S6A–D, Supporting Information). Moreover, to explore whether *ATF4* is involved in this process, *ATF4* knockdown reduced *ZnT3* mRNA expression and decreased the zinc‐induced apoptosis in *WFS1^−/−^* NPCs (Figure S7A–G, Supporting Information). Conversely, *ATF4* overexpression increased *ZnT3* mRNA expression and the zinc‐induced apoptosis in WT NPCs (Figure S7H–N, Supporting Information). These results revealed that efficacy of riluzole on mitigating zinc‐induced apoptosis is ATF4‐dependent in NPCs.

### 2.5 Riluzole Mitigates HFD‐Induced Obesity and Depression with Reduced Zinc Transportation

**Figure 5:**
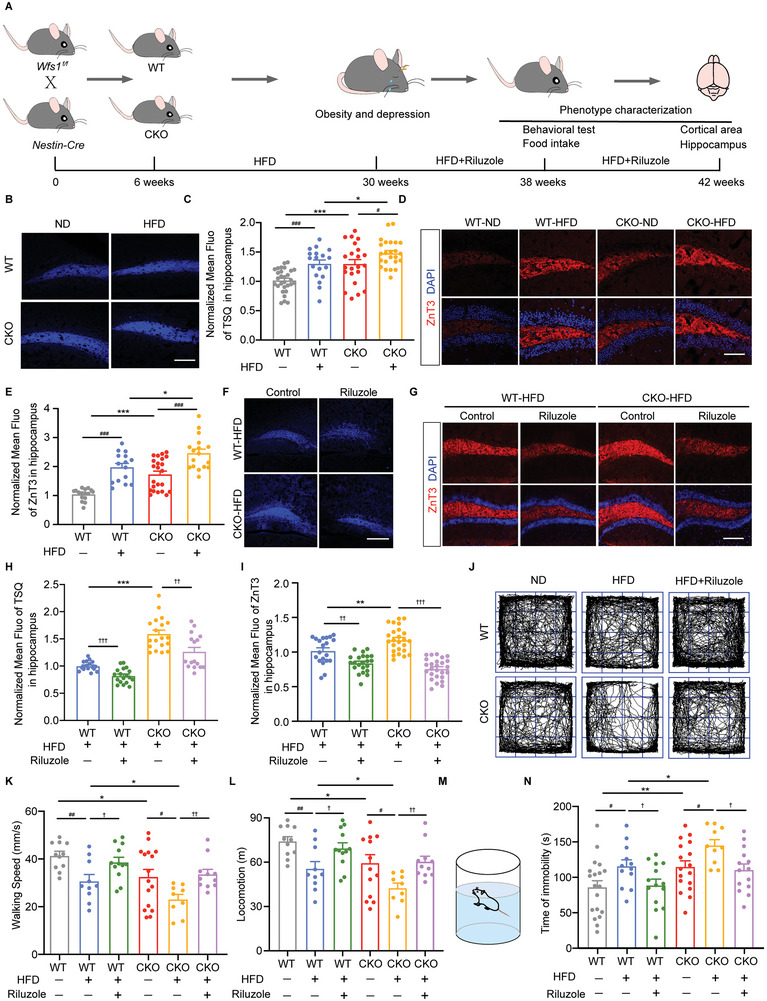
Riluzole rescues HFD‐induced depression exacerbated by neural *WFS1* deficiency. A) Scheme illustrating WT and CKO mice treated with HFD and then with riluzole or vehicle. B) Representative images of TSQ fluorescence of hippocampus in ND or HFD‐fed WT and CKO mice. Scale bar, 50 µm. C) Quantification of the normalized intensity of TSQ in the hippocampus of ND or HFD‐fed WT and CKO mice (WT + ND, *n* = 29; WT + HFD, *n* = 18; CKO + ND, *n* = 22; CKO + HFD, *n* = 24). D) Immunostaining for ZnT3 (red) and DAPI (blue) in the hippocampus of ND or HFD‐fed WT and CKO mice. Scale bar, 50 µm. E) Quantification of the normalized intensity of ZnT3 in the hippocampus of ND or HFD‐fed WT and CKO mice (WT + ND, *n* = 16; WT + HFD, *n* = 15; CKO + ND, *n* = 23; CKO + HFD, *n* = 17). F) Representative images of TSQ fluorescence in the hippocampus of HFD‐fed WT and CKO mice treated with riluzole or vehicle. Scale bar, 50 µm. G) Immunostaining for ZnT3 (Red) and DAPI (Blue) in the hippocampus of HFD‐fed WT and CKO mice treated with riluzole or vehicle. Scale bar, 50 µm. H) Quantification of the normalized intensity of TSQ in the hippocampus of HFD‐fed WT and CKO mice treated with riluzole or vehicle (WT + HFD + control, *n* = 18; WT + HFD + riluzole, *n* = 18; CKO + HFD + control, *n* = 19; CKO + HFD + riluzole, *n* = 16). I) Quantification of the normalized intensity of ZnT3 in the hippocampus of HFD‐fed WT and CKO mice treated with riluzole or vehicle (WT + HFD + control, *n* = 20; WT + HFD + riluzole, *n* = 22; CKO + HFD + Control, *n* = 25; CKO + HFD + riluzole, *n* = 26). J) Representative movement traces of ND or HFD fed WT or CKO mice treated with riluzole or vehicle in open field test. Analysis of K) the walking speed and L) the locomotion in ND or HFD‐fed WT or CKO mice treated with riluzole or vehicle in open field test(WT + ND, *n* = 11; WT + HFD + control, *n* = 10; WT + HFD + riluzole, *n* = 12; CKO + ND, *n* = 16; CKO + HFD + control, *n* = 9; CKO + HFD + riluzole, *n* = 11). M) Schematic of the forced swimming test. N) Analysis of the immobility time during the forced swimming test in ND or HFD‐fed WT or CKO mice treated with riluzole or vehicle (WT + ND, *n* = 19; WT + HFD + control, *n* = 12; WT + HFD + riluzole, *n* = 14; CKO + ND, *n* = 17; CKO + HFD + control, *n* = 10; CKO + HFD + riluzole, *n* = 14). Data were presented as mean ± SEM. Significance was calculated by unpaired two‐tailed Student's *t*‐test. ^*^ *p* < 0.05, ^**^ *p* < 0.01, and ^***^ *p* < 0.001 were considered to be significant for the comparison of WT and CKO; ^#^ *p* < 0.05, ^##^ *p* < 0.01, and ^###^ *p* < 0.001 were considered to be significant for comparison of HFD treatment; ^†^ *p* < 0.05, ^††^ *p* < 0.01, and ^†††^ *p* < 0.001 were considered to be significant for comparison of riluzole treatment.

Zinc is found in high concentrations in the hippocampus and cortex,^[^ ^45^ ^]^ consistent with our result in mice brains (Figure S8A, Supporting Information). Our preliminary results in vitro indicated that PA disturbs zinc homeostasis in NPCs and cerebral organoids. PA is an important fatty acid component of HFD, we next investigated the effect of zinc homeostasis in the nervous system of obese and depressive mice under HFD (**Figure** **5**A). TSQ staining showed that the hippocampal and cortical zinc levels were significantly elevated in HFD‐induced obese WT mice compared with ND mice, and this elevation was even more pronounced in HFD‐induced obese CKO mice (Figures 5B,C and S8B,C, Supporting Information). It has been reported that the ZnT3 protein is most abundant in the zinc‐enriched mossy fibers that project from the dentate granule cells to hilar and CA3 pyramidal neurons, and ZnT3 is responsible for the transport of zinc into the synaptic vesicle.^[^ ^46^ ^]^ Immunofluorescence data showed that ZnT3 and SYN1 were colocalized in neurons in the hippocampus DG and CA3 regions of mice (Figure S9A,B, Supporting Information). Immunostaining results showed that HFD‐induced obesity led to significant upregulation of ZnT3 in the hippocampus in both WT and CKO mice (Figure 5D,E). Since our previous results have shown that riluzole reduces the upregulation of zinc levels induced by lipotoxicity in cerebral organoids and NPCs, we next wondered whether riluzole could reduce zinc levels in mice brain. Riluzole decreased zinc levels and the apoptosis of NPCs by inhibiting *ZnT3* in the hippocampus and rescued brain size reduction in WT and CKO mice with HFD (Figures 5F–I and S10A–D, Supporting Information). Further, we sought to verify whether riluzole mitigated the aforementioned depressive‐like phenotype in HFD‐induced obese mice. To this end, we conducted behavioral evaluations of depression including the open‐field test, the forced swimming test, the elevated plus maze, and the tail suspension test. WT and CKO mice with HFD (obese model) for 6 months were then treated with 50 mg kg^−1^ per day riluzole for 2 months. Remarkably, the recovery in speed and locomotion in the open‐field test (Figure 5J–L) and the decreased immobility time in the forced swimming test (Figure 5M,N) and tail suspension test (Figure S10E,F, Supporting Information) suggested that depressive‐like behaviors were effectively mitigated by riluzole in HFD‐induced obese mice, which were more attuned to depression. Besides, riluzole alleviated depressive‐like behaviors of the obese mice, shown by an increased proportion of entries in the open arms and decreased time spent in the closed arm in the elevated plus maze test. (Figure S10G,H, Supporting Information). These findings demonstrate that riluzole could reverse depression‐like behaviors induced by HFD.

**Figure 6:**
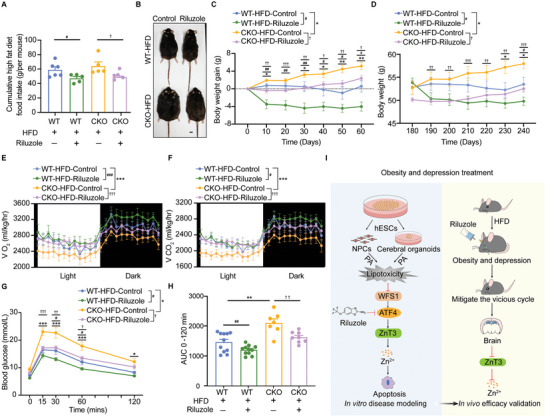
Riluzole inhibits HFD‐induced obesity aggravated by neural *WFS1* deficiency. A) Cumulative food intake in HFD‐fed WT and CKO mice treated with riluzole or vehicle (WT + HFD + control, *n* = 6; WT + HFD + riluzole, *n* = 5; CKO + HFD + control, *n* = 5; CKO + HFD + riluzole, *n* = 5). B) Representative bright‐field images of HFD‐fed WT and CKO mice treated with riluzole or vehicle. Scale bar, 5 cm. C) Body weight gain, and D) body weight trajectories in HFD‐fed WT and CKO mice treated with riluzole or vehicle (WT + HFD + control, *n* = 10; WT + HFD + riluzole, *n* = 13; CKO + HFD + control, *n* = 10; CKO + HFD + riluzole, *n* = 15). E) Oxygen consumption (VO_2_, mL/kg/h) and F) carbon dioxide production (VCO_2_, mL/kg/h) in HFD‐fed WT and CKO mice treated with riluzole or vehicle in metabolic cages over 24 h (WT + HFD + control, *n* = 7; WT + HFD + riluzole, *n* = 7; CKO + HFD + control, *n* = 6; CKO + HFD + riluzole, *n* = 7). G) IPGTT, and H) area under the curve (AUC) of IPGTT in HFD‐fed WT and CKO mice treated with riluzole or vehicle (WT + HFD + control, *n* = 11; WT + HFD + riluzole, *n* = 11; CKO + HFD + control, *n* = 7; CKO + HFD + riluzole, *n* = 8). I) The working hypothesis underlying the mechanism of neural‐specific *WFS1* bridging the vicious cycle of obesity and depression. Data were presented as mean ± SEM. Significance was calculated by A,H) unpaired two‐tailed Student's *t*‐test and C–G) two‐way ANOVA. *p < 0.05, **p < 0.01, and ***p < 0.001 were considered to be significant for comparison of WT and CKO; ^#^ *p* < 0.05, ^##^ *p* < 0.01, and ^###^ *p* < 0.001 were considered to be significant for comparison of the effect of riluzole treatment in WT. ^†^ *p* < 0.05, ^††^ *p* < 0.01, and ^†††^ *p* < 0.001 were considered to be significant for comparison of the effect of riluzole treatment in CKO.

Next, we focused on the riluzole's effects on HFD‐induced obesity. After 2 months of riluzole treatment with obese mice, WT and CKO‐HFD mice with riluzole had a decreased appetite for high‐fat food (**Figure** **6**A), significantly reduced body weight (Figure 6B–D) and elevated O_2_ consumption (VO_2_) and CO_2_ production (VCO_2_) (Figure 6E,F), suggesting riluzole mitigates the HFD‐induced obesity. Interestingly, we monitored the cumulative food intake for 32 days from 6 weeks and found both the body weight gain and food intake were attenuated in CKO‐ND mice (Figure S11A–C, Supporting Information). Our previous study reveals that *WFS1* deficiency significantly impairs synapse formation and function. *WFS1* deficiency autonomously delays neuronal differentiation and affects synapse formation.^[^ ^26^ ^]^ Previous study has shown that HFD prevents *Wfs1* mutant mice from decreasing body weight, suggesting that the *Wfs1* mutation increases the susceptibility to HFD‐induced diabetes or metabolic syndrome,^[^ ^25^ ^]^ which is consistent with our study. However, the mechanism of Wfs1‐mediated food intake‐inhibiting effects will need further evaluation. Additionally, the intraperitoneal glucose tolerance test (IPGTT) results showed that riluzole treatment mice displayed improved glucose tolerance, suggesting that riluzole could reduce the risk of diabetes by inhibiting HFD‐induced obesity (Figure 6G,H). Previous studies found that the hippocampus, cortex and hypothalamus regions of mice are associated with food intake or depression.^[^ ^47^, ^48^, ^49^, ^50^, ^51^, ^52^ ^]^ To explore *Atf4*, and *Znt3* participate in the vicious cycle of obesity and depression, we examined the mRNA expression levels of *Wfs1*, *Atf4*, and *Znt3* in the hippocampus, cortex, and hypothalamus of WT and CKO mice (Figure S12A–C, Supporting Information). CKO mice showed *Wfs1* deficiency and significantly increased expression of *Atf4* and *Znt3* in the hippocampus, cortex, and hypothalamus, suggesting the crucial role of *Wfs1* in regulation of vicious cycle of obesity and depression. Besides, ChIP results revealed the enhanced interaction of ATF4 and *Znt3* promoter by *WFS1* deficiency (Figure S12D–F, Supporting Information), suggesting that WFS1‐ATF4‐ZnT3 signaling axis regulates zinc homeostasis in mice. Collectively, these results suggest that the WFS1‐ATF4‐ZnT3 signaling axis regulates zinc homeostasis and participates in the mechanism of WFS1‐regulated association between obesity and depression and highlight the potential of riluzole as a promising approach to prevent obesity (Figure 6I).

## 3 Discussion

Depression and obesity are both major public health problems that have been extensively reported.^[^ ^4^, ^5^, ^6^ ^]^ Their vicious cycle often results in the co‐occurrence of depression and obesity, which further increases the risk of disease, but the underlying mechanisms of the vicious cycle remain poorly understood. Several studies have indicated an increased frequency of structural brain abnormalities and neuropathy in patients with obesity and T2D.^[^ ^8^, ^11^, ^12^ ^]^ *WFS1* is highly expressed in pancreatic islets and brain,^[^ ^21^, ^22^ ^]^ and its mutation is significantly associated with the risk of obesity and T2D.^[^ ^53^, ^54^, ^55^, ^56^, ^57^, ^58^, ^59^ ^]^ Meanwhile, there is a significant reduction of the brain stem in *Wfs1* ^−/−^ mice and rats.^[^ ^28^, ^29^ ^]^ Our previous research revealed that *Wfs1* loss of function in the nervous system causes depression‐like behavior in mice.^[^ ^26^ ^]^ Therefore, exploring the role of *WFS1* in brain function may represent a promising strategy for the treatment of obesity.

Cerebral organoids play a crucial role in modeling human brain development and disease.^[^ ^60^, ^61^, ^62^ ^]^ PA‐induced lipotoxicity is known to promote ER stress, autophagy, and then subsequent cell apoptosis in rat cortical cells.^[^ ^63^, ^64^ ^]^ Previous reports suggested that *Wfs1* deficiency exacerbates neurodegeneration via chronic ER stress in mice and rats.^[^ ^29^, ^57^ ^]^ Here, we used human pluripotent stem cell‐derived NPCs and cerebral organoids harboring *WFS1* deficiency with PA to mimic obesity and depression in vitro. *WFS1* deficiency heightens the susceptibility of NPCs and cerebral organoids to lipotoxicity. How *WFS1* as an ER stress regulator control PA‐induced lipotoxicity still needs to be verified in subsequent studies.

Intracellular Zn^2+^ increase is associated with neuronal cell death and brain injuries.^[^ ^30^ ^]^ Moreover, excessive Zn^2+^ accumulation has been linked to excessive lipid accumulation in the liver, leading to lipotoxicity.^[^ ^32^, ^33^ ^]^ These findings encourage us to investigate the correlation between zinc and obesity caused by lipotoxicity. Ultimately, we have verified that the deletion of *WFS1* results in apoptosis mainly through the increase of intracellular Zn^2+^ levels in 2D NPCs and 3D cerebral organoids. Our study may shed light on the novel mechanism that *WFS1* regulates lipotoxicity through intracellular Zn^2+^ in NPCs. Among a multitude of Zn^2+^ transporters (ZnTs) and Zn^2+^ importing proteins (ZIPs), *ZnT3* is expressed exclusively in the central nervous system, as a transporter receptor in synaptic vesicles.^[^ ^65^ ^]^ Our results demonstrate that *WFS1* deficiency heightens the susceptibility of neural cells to lipotoxicity and increases zinc transportation by upregulating *ZnT3* expression in vitro and in vivo. Moreover, the down‐regulation of *ZnT3* through riluzole significantly protects neural cells from lipotoxicity‐induced cell death, suggesting elevated zinc transportation as a potential pathological mechanism for the vicious cycle between obesity and depression.

Riluzole, a United States Food & Drug Administration (FDA)‐approved drug for ALS, could alleviate the neural cell death and behavior phenotype of *Wfs1* deficient mice.^[^ ^26^ ^]^ Inhibiting the glutamate release from nerve terminals as the predominant neuroprotective mechanism for riluzole target ALS patients.^[^ ^66^, ^67^ ^]^ Comorbidity exists between metabolic disorders and depressive syndrome with unclear mechanisms. It has been reported that riluzole normalizes HFD‐induced hyperactivation of glutamatergic transmissions and depression‐like behavior in mice.^[^ ^17^ ^]^ Interestingly, we showed that riluzole mitigates HFD‐induced obesity and associated depression with reduced food intake and elevated O_2_ consumption and CO_2_ production with significant inhibition of ZnT3 expression in HFD mice (Figures 6A–F and 5G, H). *Wfs1* deficient CKO mice significantly increased expression of *Atf4* and *Znt3* in the hippocampus, cortex and hypothalamus (Figure S12A‐C, Supporting Information), suggesting that WFS1‐ZnT3‐Zn^2+^ axis regulates the vicious cycle of obesity and depression.  hypothalamus plays an essential role in controlling food intake and energetic status.^[^ ^68^, ^69^ ^]^ Hypothalamic pro‐opiomelanocortin (POMC) neurons in hypothalamus have been reported to play a crucial role in regulating energy homeostasis and glucose/lipid metabolism. Somatostatin‐positive (SST^+^) GABAergic neurons in the hypothalamus and D1 medium spiny neurons in the striatum have been reported to be associated with food motivation.^[^ ^70^, ^71^ ^]^ Thus, systemically administered riluzole has the potential to alleviate depressive‐like behaviors with increased metabolic rates by functioning within hippocampus, cortex, and hypothalamus, which warrants further evaluation.

## 4 Conclusion

In summary, this study revealed the role of neural‐specific *WFS1* deficiency in exacerbating the vicious cycle of obesity and depression in HFD mice. *WFS1* deficiency in the nervous system exacerbates HFD‐induced obesity and depressive‐like behaviors. *WFS1* deficiency increased lipotoxicity‐induced apoptosis of neural precursor cells in human cerebral organoids and 2D NPCs via regulating zinc homeostasis by the WFS1‐ATF4‐ZnT3 signaling axis. The exacerbation of obesity and depression‐like behaviors in HFD‐induced obese mice due to *WFS1* deficiency was effectively relieved by riluzole. Thus, maintaining zinc homeostasis in the neural system could serve as a valuable tool to combat the vicious cycle of obesity and depression.

## 5 Experimental Section

### Ethical Statement

All animal experiments were approved by the guidelines of the Institute of Laboratory Animal Research Center, Tongji University. The approval number for these experiments was TJAB03621103.

### Plasmid Constructs and Materials

pWPI‐EGFP empty vector was used to construct the *WFS1* overexpression plasmids constructs. The *WFS1* overexpression construct was generated using the Gateway system (Invitrogen) by PCR amplifying *WFS1* from the human pancreas. pENTER‐mCherry empty vector was used to construct the *ATF4* overexpression plasmids construct. *ZnT3* or *ATF4* short hairpin RNA (shRNA) constructs were designed to target specific 21‐base regions of *ZnT3* or *ATF4* and then cloned into the pLKO.1‐TRC plasmid. The primer used for *ZnT3* shRNA is 5′‐CTGTGCCGTTTGCTTTGTCTT‐3′. The primer used for *ATF4* shRNA is 5′‐ GCCTAGGTCTCTTAGATGATT‐3′. CRISPR‐Cas9‐mediated *WFS1* knockout constructs were generated as described previously.^[^ ^26^ ^]^

### Generation of WFS1 Knockout hESCs via CRISPR/Cas9

Feeder‐free hESCs (HuES8 and H1 lines) were cultured on Matrigel‐coated dishes with mTeSR (Stemcell Technologies). According to the previous study, one or two sgRNAs targeting one or two different loci on the *WFS1* gene were cloned into P2U6 vectors. hESCs were transfected with the P2U6 plasmid carrying Cas9 and sgRNAs corresponding to the loci of *WFS1* gene. After electroporation, the hESCs were added 40 µg mL^−1^ puromycin for 2 d for selection. PCR was used to validate the knock‐out efficiency. An extra nucleotide G was introduced at 70 bp on the 2nd exon of *WFS1*, leading to frame‐shift mutation and an early stop codon in the transcript.

### Human Cerebral Organoid Generation

Cerebral organoids were generated according to previously published methods.^[^ ^60^ ^]^ Briefly, H1 hESCs were dissociated into single cells using accutase (Stemcell Technologies). 9000 cells were plated in each well of an ultra‐low binding 96‐well plate (Corning) with mTeSR medium including 5 × 10^−6^ m ROCK inhibitor (RI, Y‐27632; Stemcell Technologies) to form EBs. RI was removed on Day 1. EBs were cultured in neural induction media (NI media) containing DMEM/F12 (Gibco), 1:100 N2‐supplement (Gibco), GlutaMAX (Gibco), MEM‐NEAA (Sigma), penicillin/streptomycin (Gibco) and 1 µg mL^−1^ heparin (Sigma) to form neuroepithelial tissues on Day 5. NI medium was changed every other day for 5 d. On Day 11, EBs were embedded in droplets of cold Matrigel (Corning) and transferred to a 10 cm dish with differentiation medium without vitamin A containing 1:1 mixture of DMEM/F12 and Neurobasal (Gibco) medium, 1:100 (B27‐supplement without vitamin A (Gibco), GlutaMAX, penicillin/streptomycin), 1:200 (N2‐supplement and MEM‐NEAA), 1:4000 insulin (Sigma) and 1 mg mL^−1^ NaHCO_3_. EB droplets were transferred to the orbital shaker (57 r.p.m.) on Day 16. The differentiation medium without vitamin A was replaced by a differentiation medium with vitamin A containing B27‐supplement with vitamin A (Gibco), 70 µg mL^−1^ vitamin C (Sigma), and other reagents components same as the above medium on Day 21. The differentiation medium was changed once in 5 days. 2% Matrigel was added to the differentiation medium with vitamin A from Day 40.

### Human Neural Progenitor Cell Generation

Feeder‐free hESCs (HuES8 lines) were cultured on Matrigel‐coated dishes with mTeSR for 7 d. Subsequently, mTeSR medium was substituted by N2 medium (DMEM/F12 supplemented with 0.5 × N2‐Supplement, 1 × 10^−6^ m dorsomorphin, and 1 × 10^−6^ m SB431542) for 2 d. hESCs colonies were lifted off and suspension cultured with N2 medium on the shaker (95 r.p.m.) for 7 d to form EBs. Then EBs were mechanically dissociated and cultured on 10 µg mL^−1^ poly‐L‐ornithine (Sigma) and laminin (Gibco) coated dishes with N2B27 medium (DMEM/F12 supplemented with 0.5 × N2‐Supplement, 0.5 × B27‐Supplement and 20 ng mL^−1^ bFGF (Stemcell Technologies)) to form neural rosettes. The neural rosettes were manually collected, dissociated using accutase into single NPCs, transferred to poly‐L‐ornithine/laminin‐coated dishes, and cultured in an N2B27 medium.

### Animals, Diets, and Drug Intervention in Mice

Using the CRISPR‐Cas9 system, *Wfs1* ^flox/flox^ mice were obtained from Shanghai Model Organisms Center, Inc. (Shanghai, China). To obtain *Wfs1* conditional knockout (CKO) mice, *Wfs1* ^flox/flox^ mice were crossed with *Nestin‐Cre* mice. All mice were housed under a 12 h light/dark cycle in Laboratory Animal Research Center, Tongji University.

The experimental mice were randomly divided into four groups as follows: normal diet *Wfs1* ^flox/flox^ mice (WT‐ND), high‐fat diet *Wfs1* ^flox/flox^ mice (WT‐HFD), normal diet *Wfs1* ^flox/flox^; *Nestin‐Cre* mice (CKO‐ND), and high‐fat diet *Wfs1* ^flox/flox^; *Nestin‐Cre* mice (CKO‐HFD). As a drug intervention, HFD‐fed WT and CKO mice were treated with 50 mg kg^−1^ per day riluzole in drinking water for 2 months from 6 months to 8 months. The mice in the control group drink water normally.

### Behavioral Tests—Open field test

OFT was used to evaluate spontaneous locomotor activity and exploratory behavior.^[^ ^72^ ^]^ The apparatus consisted of a cubic black box (60 cm x 60 cm x 60 cm), a center square area (30 cm x 30 cm), a video camera, and an analysis system. Mice were placed at the center of the center square area and allowed to explore freely for 30 min. The bottom and inner walls of the center box were cleaned after each session to remove olfactory cues. The motion speed, movement distance, and immobility were calculated through software. The entire experiment was conducted and recorded throughout in a quiet environment.

### Forced Swimming Test

Mice were placed into a cylinder (15 cm diameter, 20 cm height) filled with water. The water temperature was 22–24 °C, and the water depth was 13 cm above the bottom to prevent the mouse from touching the cylinder bottom. The entire test lasted for 6 min. The immobility time of the mice during the testing period was recorded after the initial 2 min. Immobility time was recorded when mice stopped struggling for ≥1 s or passively floating on the water (moving lightly to keep balance). The entire experiment was conducted and recorded throughout in a quiet environment.

### Tail Suspension Test

The test apparatus consisted of a blue box (40 cm × 40 cm × 60 cm), with one open wall in which mice could be video recorded. The apparatus could be separated into two parts using an opaque partition, and two mice could be tested simultaneously. The mouse was suspended and the tip of the tail <1 cm was fixed onto the instrument with adhesive tape. The head of the mouse was 15 cm above the ground. The entire test lasted for 6 min. The immobility time of the mice was recorded after the initial 2 min and recorded when mice stopped struggling for ≥1 s. The entire experiment was conducted and recorded throughout in a quiet environment.

### Elevated Plus Maze Test

The elevated plus maze test equipment consisted of a central square (10 cm × 10 cm) and four arms (45 cm long × 10 cm wide, two open arms with no railing, and two closed arms enclosed by a transverse wall 15 cm in height). The height of the maze was 80 cm above the floor. Briefly, each mouse was placed in the central square, faced the open arm, and allowed to explore the maze for 10 mins. The entry number of the mice crossed the open and closed arms and the amount of time spent in the open arm and closed arm was recorded as previously described.^[^ ^73^ ^]^

### Quantitative Real‐Time PCR (qPCR)

Primer 5.0 software was used for designing for the qPCR primers of target genes. TRNzol (Tiangen) and FastQuant RT Kit (Tiangen) were used for total RNA extraction and cDNA reverse transcription, respectively. Quantitative real‐time PCR was performed using SuperReal PreMix Plus (SYBR Green) (Tiangen) on Lightcycler 96 (Roche). The amplification efficiency for each primer and the cycle threshold (Ct) were determined automatically by Lightcycler software (Roche). The fold‐change was calculated by the comparative CT (2^−ΔΔCT^) method against GAPDH. The primers used in this experiment were shown in the Tables S1 and S2 (Supporting Information).

### Immunofluorescence Staining

For mouse brain and human cerebral organoids, slides were first blocked with PBST (PBS with 0.1% TritonX‐100) containing 5% donkey serum for 1 h. For cell immunofluorescence, the cells were fixed with 4% paraformaldehyde (PFA) for 15 min at room temperature (RT), washed three times with PBS, and then blocked with PBST containing 5% donkey serum for 15 min. Incubation with primary antibodies was performed overnight at 4 °C. The primary antibodies used in this article were as follows: SOX2 (1:1000, R&D, AF2018), CAS3 (1:200, Cell Signaling Technology, 9664S), MAP2 (1:500, Abcam, ab5392), ZnT3 (1:500, Synaptic Systems, 197002), WFS1(1:100, Abcam, ab259362), Synapsin 1 (1:500, Synaptic systems, 106001), PSD95 (1:500, Abcam, ab18258), BDNF (1:500, Abcam, ab108319), Nestin (1:10, DSHB, Rat‐401). After washing three times with PBST, the secondary antibodies were then applied for 1 h at RT. Images were captured with a Zeiss Axio Imager M2 fluorescence microscope and a Leica SP8 confocal microscope. Fluorescence images were analyzed using ImageJ version 1.52.

### IPGTT

Mice were fasted for 16 h before the test. Tail vein blood samples were collected. The basal glucose level was determined as a starting point, glucose (1 g kg^−1^ body weight) was injected intraperitoneally, and plasma glucose levels were measured 15, 30, and 60 min after injection.

### Indirect Calorimetry

Oxygen consumption (VO_2_) and carbon dioxide production (VCO_2_) were measured in a subgroup of mice using a Comprehensive Lab Animal Monitoring System (CLAMS) (Columbus Instruments, Columbus, OH). Briefly, individual mice with ad libitum access to food and water were acclimatized to metabolic cages for 12 h before 3 d of continuous recording, with data collected every 8 min. The O_2_ and CO_2_ content of the sample air from each cage was determined using an open‐circuit Oxymax system, which passed the air through sensors to measure oxygen and carbon dioxide levels.

### Flow Cytometry‐Annexin V/PI Apoptosis Analysis

NPCs were treated with PA (Sigma‐Aldrich, #P0500) or control (8% BSA in 0.1 m NaOH) in NGF medium for 48 h and dissociated into single cells using accutase. Cell death was measured with an Annexin V/PI apoptosis detection kit (YEASEN, 40304ES60) and assessed by flow cytometry.

### Zn^2+^ Analysis

NPCs were dissociated into single cells using accutase as described above and incubated with Zinquin (20 × 10^−9^ m, Sigma‐Aldrich, #Z2251) diluted in FACS buffer for 25 min at 37 °C and 5% CO_2_. Cells were washed and resuspended in FACS Buffer and then analyzed by CytoFLEXLX (Beckman Coulter). Data analysis was performed using FloJo version 10.4.

### TSQ Staining

TSQ Staining was performed as previously described.^[^ ^74^, ^75^, ^76^ ^]^ The unfixed frozen brains were sectioned coronally at 14 µm thick. Sections were immersed in a solution of 4.5 × 10^−6^ m TSQ for 60 s and then washed in 0.9% normal saline for 60 s. TSQ binding was examined using DAPI filter settings with a fluorescence microscope (Zeiss LSM 880 or Leica SP8). TSQ‐stained area and intensity were measured using ImageJ version 1.52.

### Zinquin Staining

NPCs were washed once with DPBS and then incubated with Zinquin (20 × 10^−6^ m, Sigma‐Aldrich, #Z2251) diluted in PBS containing 0.5% BSA for 25 min at 37 °C and 5% CO_2_. Zinquin‐stained NPCs were washed in 0.5% BSA and examined using a Zeiss microscope with emission at 384 nm and excitation at 364 nm.

### Chromatin Immunoprecipitation (ChIP)‐PCR

*For mouse brain*: First, approximately 50 mg of mouse brain cortex was crushed into powder in liquid nitrogen. Liver powders were transferred into centrifuge tubes and incubated with newly prepared 1% formaldehyde in 10 mL PBS for 10 min at RT. Crosslinking was quenched by adding 2.5 m glycine to a final concentration of 0.125 m glycine and incubation for 5 min at RT. The crosslinked samples were centrifuged at 800 *g* for 5 min. The supernatant was discarded, and the pellet was washed twice with PBS followed by resuspension in 500 µL cold cell lysis buffer (RIPA buffer supplemented with a cocktail of protease inhibitors), and homogenized with Dounce homogenizer (60 HZ, 1 min). The homogenate was then centrifuged at 800 *g* for 5 min at 4 °C and the supernatant was collected.

### For cells

NPCs were treated with 5 × 10^−6^ m ZnPTO or vehicle for 3 h and then digested using accutase. One million NPCs were incubated with 1% formaldehyde with gentle shaking for 10 min at RT, and crosslinking was stopped by the addition of 2.5 m glycine to a final concentration of 0.125 m glycine. After two washes with cold PBS containing protease inhibitors, cells were pelleted by centrifugation and resuspended in lysis buffer (RIPA buffer supplemented with a cocktail of protease inhibitors).

Fragments of the genomic DNA were broken using Covaris. After antibody‐coupled Dynabeads centrifugation, the supernatant was diluted in ChIP dilution buffer and incubated overnight at 4 °C with anti‐ATF4 antibody (Protientech,10835‐1‐AP) and anti‐IgG (Cell signaling, #7074S). Samples were washed two times in RIPA buffer, one time in TE buffer and then resuspended in elution buffer with proteinase K at 68 °C for 2 h. DNA was purified using a DNA purification spin column (Qiagen, 129056) and dissolved in nuclease‐free water. The immunoprecipitated DNA was quantified using qPCR, and all values were normalized to the input. The primers used in this experiment are shown in Tables S3 and S4 (Supporting Information).

### Western Blot

NPC cells were collected, washed with PBS, and lysed in RIPA buffer with protease inhibitors. Protein concentration was measured with a BCA Protein Assay Reagent Kit (YEASEN, #20201ES76). Equal amounts of protein were separated on 8% SDS‐PAGE and transferred to nitrocellulose membranes (PALL, #66485), which were probed with antibodies against ATF4 (1:1000, Protientech, 10835‐1‐AP), or antibodies against GAPDH (1:5000, Abways, AB0037) overnight at 4 °C. After washing, the membranes were then probed with Peroxidase AffiniPure Goat Anti‐Rabbit lgG (H+L) (YEASEN, #33101ES60) at RT for 1 h. Finally, the images were acquired with Amersham Imager 600 and quantified with ImageJ version 1.52. All western blot results were provided as representative images from three independent experiments.

### Statistics

The experiments were carried out with at least three independent biological replicates, and all values were shown as the mean ± SEM. Statistical significance between two groups was calculated by unpaired two‐tailed Student's *t*‐test, and between three or more groups was calculated by two‐way ANOVA followed by Bonferroni's post hoc correction. Statistically significant differences were considered to be ^*^ *p* < 0.05, ^**^ *p* < 0.01, ^***^ *p* < 0.001, ^****^ *p* < 0.0001. n.s. not significant.

## Conflict of Interest

The authors declare no conflict of interest.

## Author Contributions

M.G. and Y.F. contributed equally to this work. M.G., Y.F., Z.‐N.Z, and W.L. conceived and designed the experiments. M.G., Y.F., and K.Y. performed the experiments and analyzed the data. F.Y. and R.H. helped to establish the human cerebral organoid culture system. F.Y., J.C., and R.Z. guided the behavioral tests. W.X. and Q.M. helped to construct the ATF4 overexpression plasmid and virus packaging. Y.S. and Y.Y. provided useful suggestions for the project. M.G., Z.‐N.Z, K.Y., Y.F., Y.Y, and W.L. wrote the manuscript.

## Supporting information



Supporting Information

## Data Availability

Data sharing is not applicable to this article as no new data were created or analyzed in this study.
