# Supplementary material for: The WFS1‐ZnT3‐Zn2+ Axis Regulates the Vicious Cycle of Obesity and Depression
Source: Adv Sci (Weinh). 2024 Sep 11;11(41):2403405. doi: 10.1002/advs.202403405 (PMC11538679; doi:10.1002/advs.202403405)
Supplement: Supplementary file 1 — Supporting Information [file ADVS-11-2403405-s001.pdf]

ADVANCED SCIENCE
Open Access

Supporting Information

for *Adv. Sci.*, DOI 10.1002/advs.202403405

The WFS1-ZnT3-Zn^{2+} Axis Regulates the Vicious Cycle of Obesity and Depression

Mengting Gong, Yulin Fang, Kaijiang Yang, Fei Yuan, Rui Hu, Yajuan Su, Yiling Yang, Wenjun Xu, Qing Ma, Jiaxue Cha, Ru Zhang, Zhen-Ning Zhang and Weida Li**

Supplementary Materials

Title

The WFS1-ZnT3-Zn^{2+} axis regulates the vicious cycle of obesity and depression

Authors

Mengting Gong [1#], Yulin Fang [1#], Kaijiang Yang [1], Fei Yuan [1], Rui Hu [1], Yajuan Su [1], Yiling Yang [1], Wenjun Xu[1], Qing Ma[1], Jiaxue Cha [2], Ru Zhang [2], Zhen-Ning Zhang [1*], Weida Li [1*]

Affiliations

[1] Institute for Regenerative Medicine, State Key Laboratory of Cardiology and Medical Innovation Center, Shanghai East Hospital, Frontier Science Center for Stem Cell Research, Shanghai Key Laboratory of Signaling and Disease Research, School of Life Sciences and Technology, Tongji University, Shanghai 200092, China.

[2] Shanghai Key Laboratory of Signaling and Disease Research, School of Life Sciences and Technology, Tongji University, Shanghai 200092, China.

[#]These authors contributed equally: Mengting Gong, Yulin Fang.

*Correspondence author

E-mail: znzhang@tongji.edu.cn, weidali@tongji.edu.cn.

Further information and requests for resources and reagents should be directed to and will be fulfilled by the lead contact, weidali@tongji.edu.cn.

Supplemental figure and table information

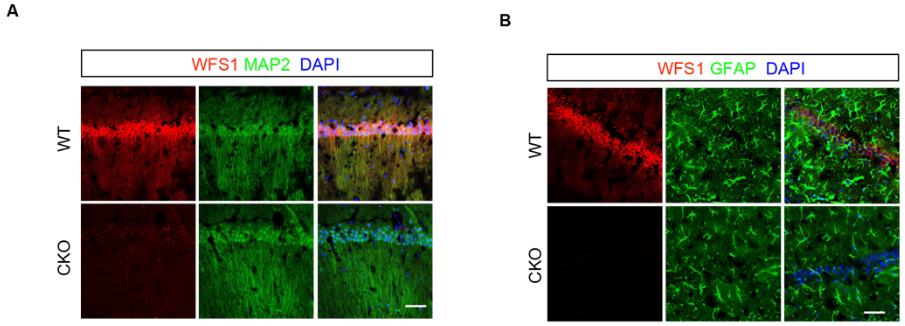

Figure. S1. Specific deficiency of WFS1 in neurons and astrocytes from CKO mice

A) Immunostaining for WFS1 (Red), MAP2 (Green), and DAPI (Blue) in the hippocampus of WT and CKO mice. Scale bar, 50 µm. B) Immunostaining for WFS1 (Red), GFAP (Green), and DAPI (Blue) in the hippocampus of WT and CKO mice. Scale bar, 50 µm.

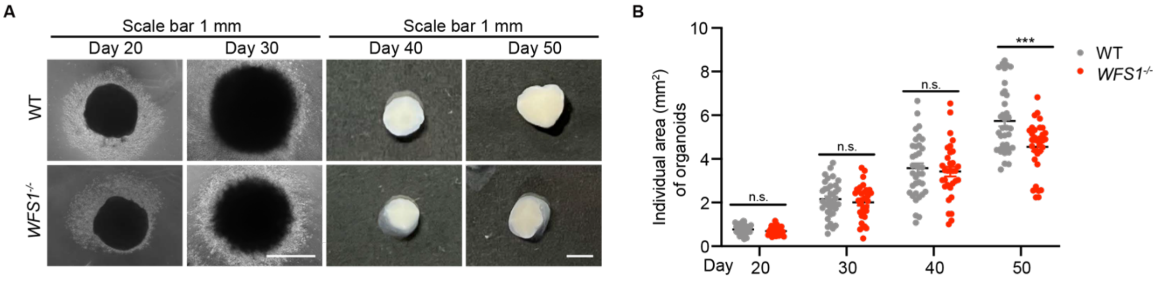

Figure. S2. *WFS1* deficiency reduces the size of human cerebral organoids

A) Representative bright-field images of WT and *WFS1*$^{-/-}$ cerebral organoids at Day 20, Day 30, Day 40, and Day 50. Scale bar, 1 mm. B) Quantification of the individual area (mm^2) of WT and *WFS1*$^{-/-}$ cerebral organoids at Day 20, Day 30, Day 40, and Day 50 (WT, n = 36; *WFS1*$^{-/-}$, n = 32). Data were presented as mean ± SEM. Significance was calculated by unpaired two-tailed Student's *t*-test. ***$p < 0.001$ was considered to be significant.

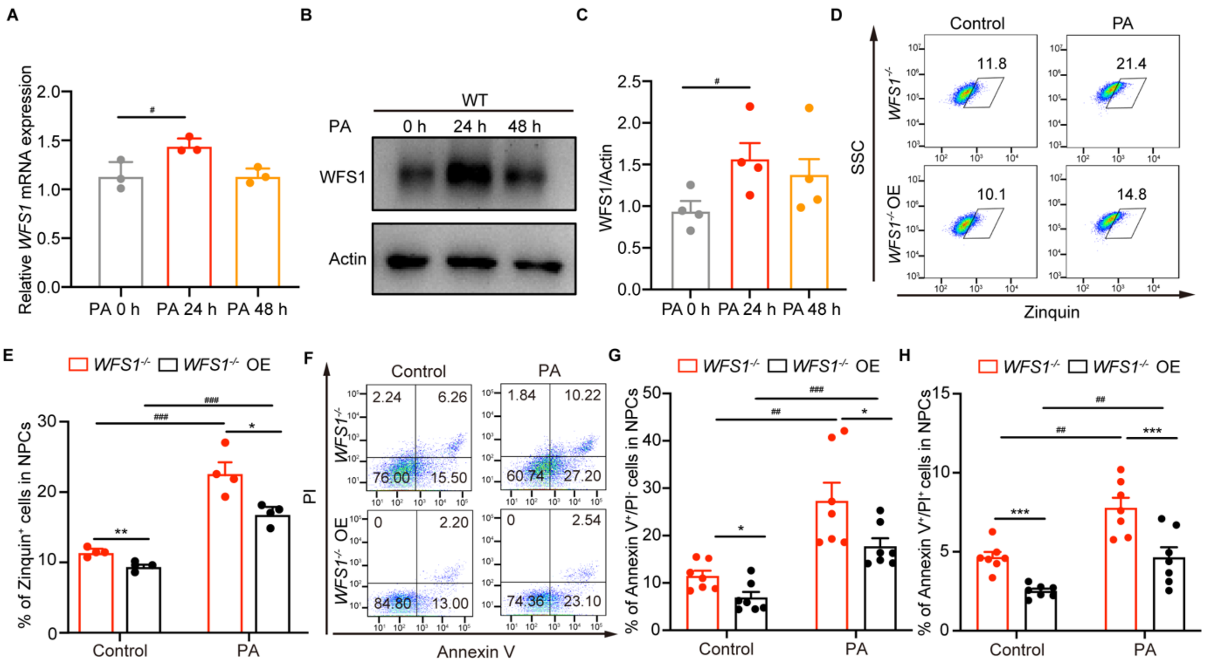

Figure. S3. *WFS1* rescues lipotoxicity-induced apoptosis by regulating zinc homeostasis in NPCs

A) Quantitative real-time PCR analysis of *WFS1* expression in NPCs treated with PA at 0 h, 24 h, and 48 h (n = 3). B-C) Representative immunoblots (B) and quantification (C) of WFS1 and GAPDH in WT NPCs treated with PA at 0 h, 24 h, and 48 h (n = 4). D-E) Representative FACS plots (D) and quantification (E) of the percentage of Zinquin$^+$ cells in *WFS1*$^{-/-}$ NPCs infected with WFS1 OE treated with PA for 48 h (n = 4). F-H) Representative FACS plots (F) and quantification of death cells (G, Annexin V$^+$/PI$^-$; H, Annexin V$^+$/PI$^+$) in *WFS1*$^{-/-}$ NPCs infected with WFS1 OE treated with PA for 48 h by double-staining with Annexin V and PI (n = 7). Data were presented as mean ± SEM. Significance was calculated by unpaired two-tailed Student's *t*-test. $^{*}p < 0.05$, $^{**}p < 0.01$, and $^{***}p < 0.001$ were considered to be significant for comparison of *WFS1*$^{-/-}$ and *WFS1*$^{-/-}$ OE; $^{\#}p < 0.05$, $^{\#\#}p < 0.01$, and $^{\#\#\#}p < 0.001$ were considered to be significant for comparison of PA treatment.

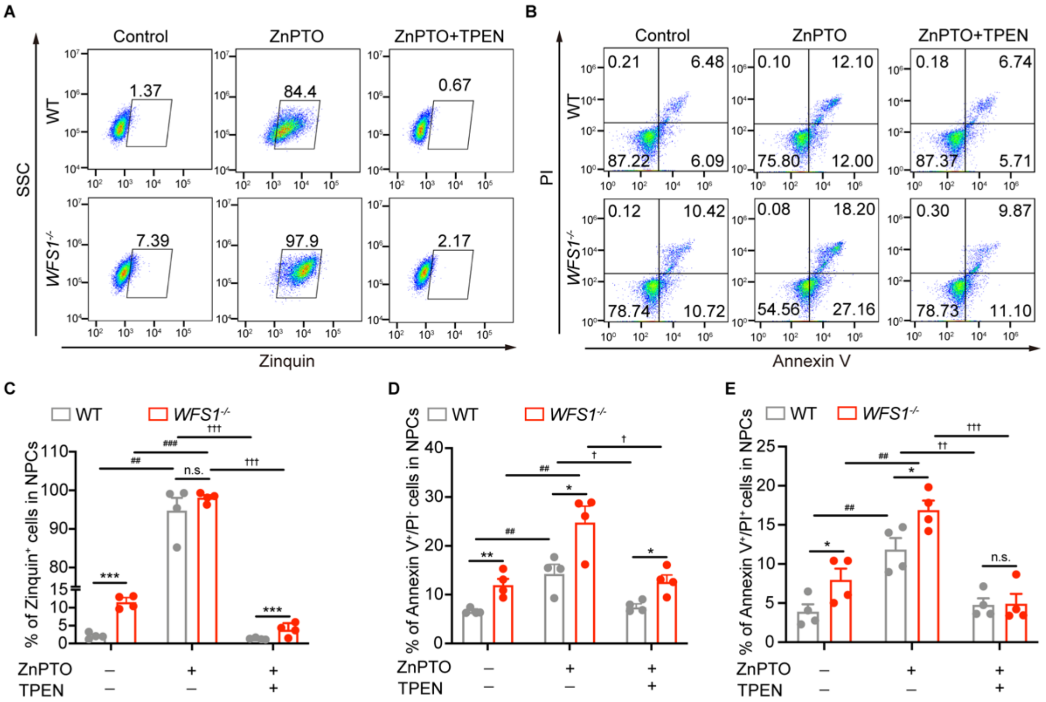

Figure. S4. ***WFS1* deficiency exacerbates zinc-induced apoptosis in NPCs**

A) Representative FACS plots of WT and *WFS1*$^{-/-}$ NPCs treated with ZnPTO or ZnPTO and TPEN by Zinquin staining. B) Representative FACS plots of WT and *WFS1*$^{-/-}$ NPCs treated with ZnPTO or ZnPTO and TPEN by double-staining with Annexin V and PI. C) Quantification of the percentage of Zinquin$^+$ cells in WT and *WFS1*$^{-/-}$ NPCs treated with ZnPTO or ZnPTO and TPEN (n = 4). D-E) Quantification of death cells (D, Annexin V$^+$/PI$^-$; E, Annexin V$^+$/PI$^+$) in WT and *WFS1*$^{-/-}$ NPCs treated with ZnPTO or ZnPTO and TPEN (n = 4). Data were presented as mean ± SEM. Significance was calculated by unpaired two-tailed Student's *t*-test. $^{*}p < 0.05$, $^{**}p < 0.01$, and $^{***}p < 0.001$ were considered to be significant for comparison of WT and *WFS1*$^{-/-}$; $^{\#}p < 0.05$, $^{\#\#}p < 0.01$, and $^{\#\#\#}p < 0.001$ were considered to be significant for comparison of ZnPTO treatment; $^{\dagger}p < 0.05$, $^{\dagger\dagger}p < 0.01$, and $^{\dagger\dagger\dagger}p < 0.001$ were considered to be significant for comparison of ZnPTO and TPEN treatment.

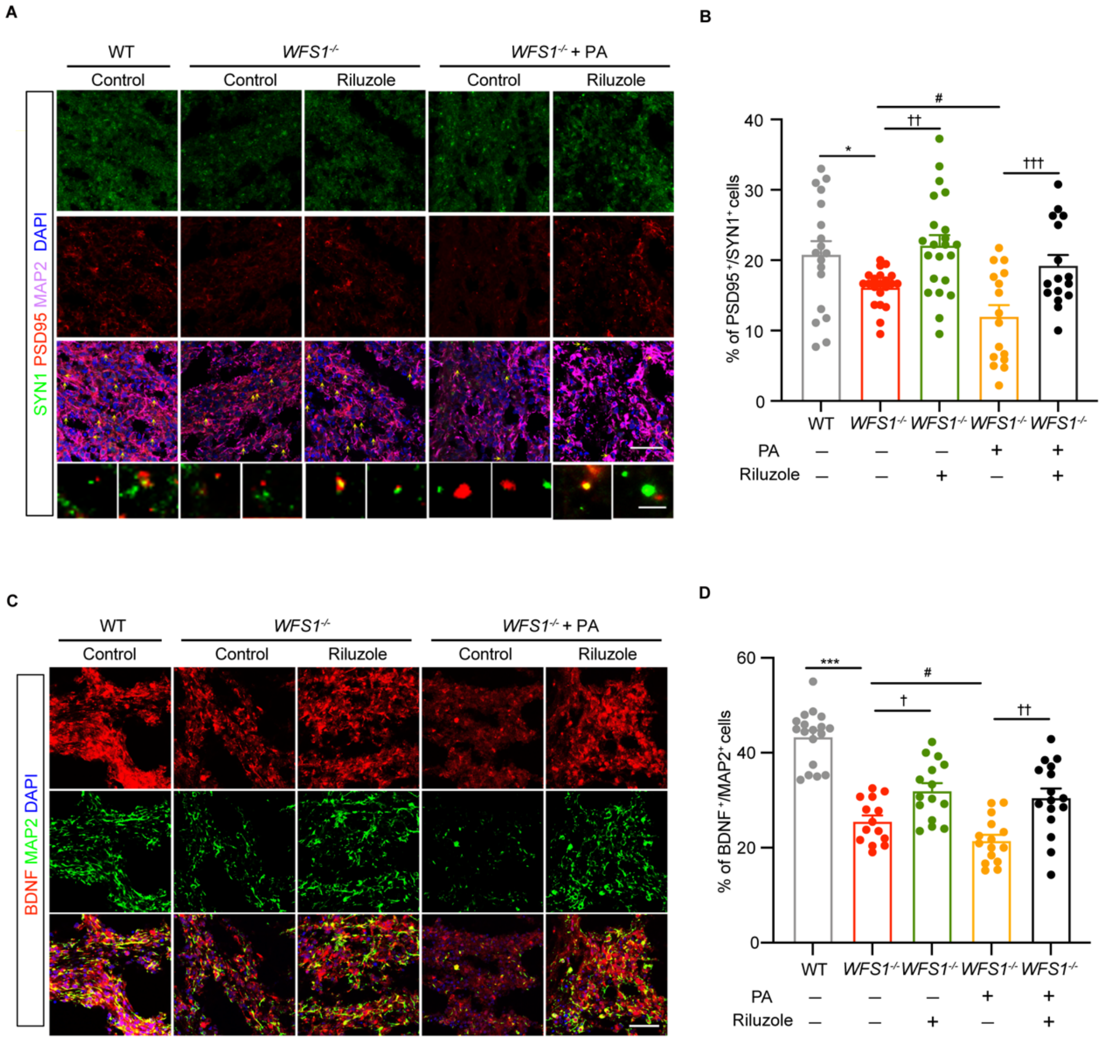

Figure. S5. Riluzole rescues the synapse formation in *WFS1*$^{-/-}$ cerebral organoids

A) Immunostaining for SYN1(Green), PSD95 (Red), MAP2 (Magenta), and DAPI (Blue) in WT and *WFS1*$^{-/-}$cerebral organoids treated with vehicle or 1 mM PA and 5 µM riluzole for 5 days. Scale bar, 50 µm. B) Quantification of the percentage of PSD95^{+} cells among the total number of SYN1^{+} cells in WT and *WFS1*$^{-/-}$ cerebral organoids treated with vehicle or 1 mM PA and 5 µM riluzole for 5 days. (WT + Control, n = 18; *WFS1*$^{-/-}$ + Control, n = 21; *WFS1*$^{-/-}$ + Riluzole, n = 21; *WFS1*$^{-/-}$ + PA + Control, n = 16; *WFS1*$^{-/-}$ + PA + riluzole, n = 16). C) Immunostaining for BDNF (Red), MAP2 (Green), and DAPI (Blue) in WT and *WFS1*$^{-/-}$ cerebral organoids treated with vehicle or 1 mM PA and 5 µM riluzole for 5 days. Scale bar, 50 µm. D) Quantification of the percentage of BDNF^{+} cells among the total number of

MAP2$^+$ cells in WT and *WFS1*$^{-/-}$ cerebral organoids treated with vehicle or 1 mM PA and 5 μM riluzole for 5 days. (WT + Control, n = 18; *WFS1*$^{-/-}$ + Control, n = 14; *WFS1*$^{-/-}$ + riluzole, n = 15; *WFS1*$^{-/-}$ + PA + Control, n = 15; *WFS1*$^{-/-}$ + PA + riluzole, n = 16). Data were presented as mean ± SEM. Significance was calculated by unpaired two-tailed Student's *t*-test. *$p < 0.05$, **$p < 0.01$, and ***$p < 0.001$ were considered to be significant for comparison of WT and *WFS1*$^{-/-}$; $^{\#}p < 0.05$, $^{\#\#}p < 0.01$, and $^{\#\#\#}p < 0.001$ were considered to be significant for comparison of PA treatment; $^{\dagger}p < 0.05$, $^{\dagger\dagger}p < 0.01$, and $^{\dagger\dagger\dagger}p < 0.001$ were considered to be significant for comparison of riluzole treatment.

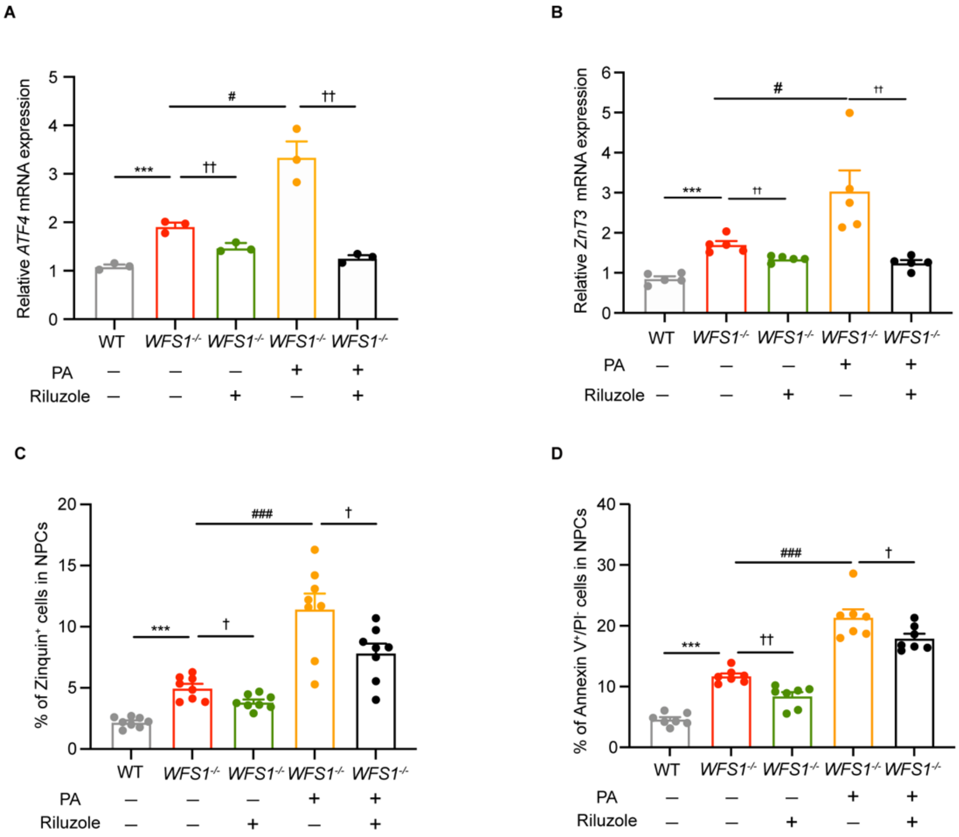

Figure. S6. Riluzole reduces apoptosis by regulating zinc homeostasis *via* the WFS1-ATF4-ZnT3 signaling axis in NPCs

A) Quantitative real-time PCR analysis of *ATF4* in WT and *WFS1*$^{-/-}$ NPCs treated with PA or vehicle and riluzole for 48 h (n = 3). B) Quantitative real-time PCR analysis of *ZnT3* in WT and *WFS1*$^{-/-}$ NPCs treated with PA or vehicle and riluzole for 48 h (n = 5). C) Quantification of the percentage of Zinquin$^+$ cells in WT and *WFS1*$^{-/-}$ NPCs treated with PA

or vehicle and riluzole for 48 h (n = 8). D) Quantification of Annexin V^{+}/PI^{-} cells in WT and *WFS1*$^{-/-}$ NPCs treated with PA or vehicle and riluzole for 48 h (n = 7). Data were presented as mean ± SEM. Significance was calculated by two-tailed Student's *t*-test. $^{*}p < 0.05$, $^{**}p < 0.01$, and $^{***}p < 0.001$ were considered to be significant for comparison of WT and *WFS1*$^{-/-}$; $^{\#}p < 0.05$, $^{\#\#}p < 0.01$, and $^{\#\#\#}p < 0.001$ were considered to be significant for comparison of PA treatment; $^{\dagger}p < 0.05$, $^{\dagger\dagger}p < 0.01$, and $^{\dagger\dagger\dagger}p < 0.001$ were considered to be significant for comparison of riluzole treatment.

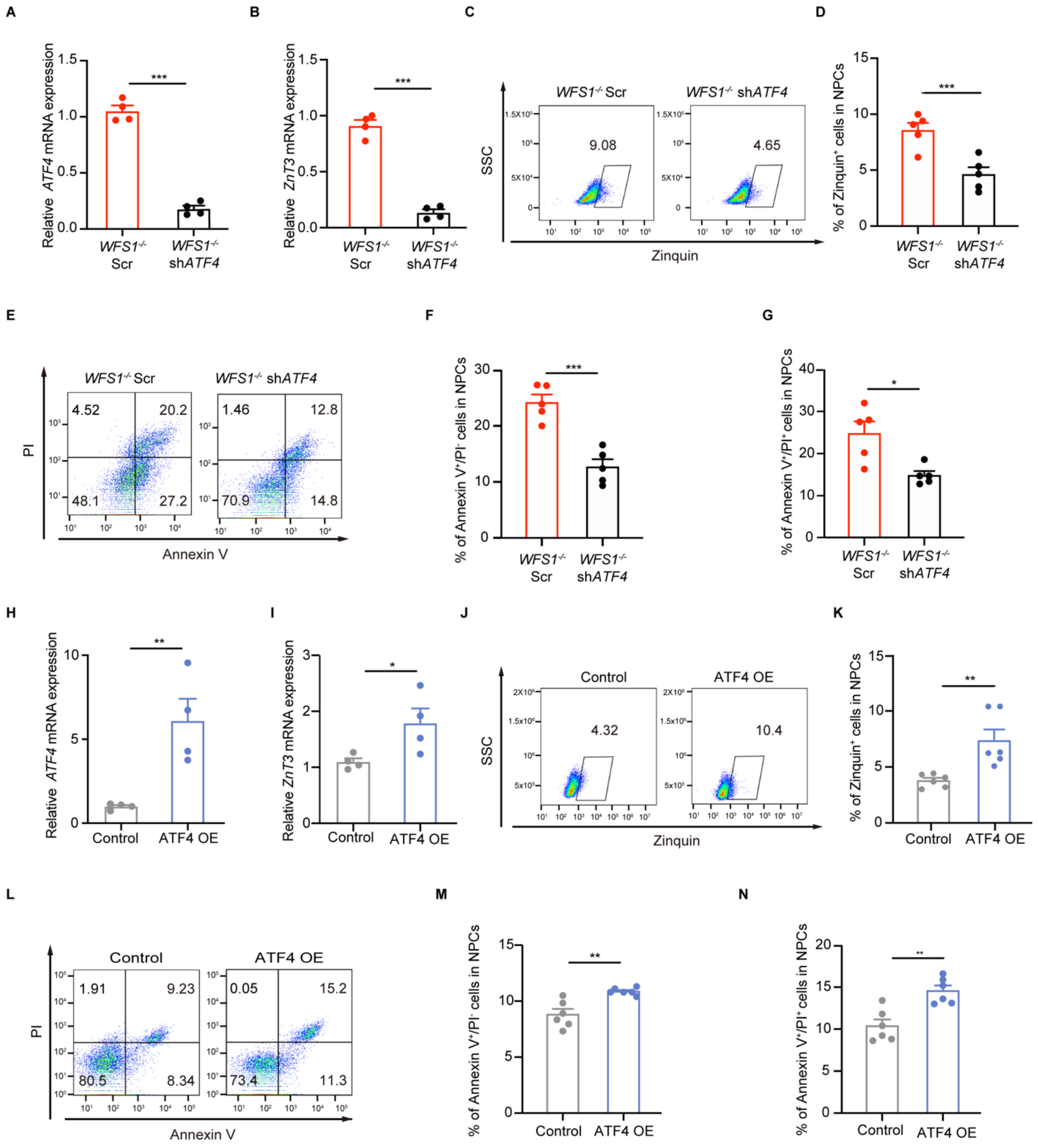

Figure. S7. ATF4 induces elevated zinc and apoptosis in NPCs

A) Quantitative real-time PCR analysis of *ATF4* in *WFS1*$^{-/-}$ NPCs infected with Lenti-shATF4 or Scr (n = 4). B) Quantitative real-time PCR analysis of *ZnT3* in *WFS1*$^{-/-}$ NPCs infected with Lenti-shATF4 or Scr (n = 4). C-D) Representative FACS plots (C) and quantification (D) of the percentage of Zinquin^{+} cells in *WFS1*$^{-/-}$ NPCs infected with Lenti-

shATF4 or Scr (n = 5). E-G) Representative FACS plots (E) and quantifications of death cells (F, Annexin V^{+}/PI^{-}; G, Annexin V^{+}/PI^{+}) of *WFS1*$^{-/-}$ NPCs infected with Lenti-shATF4 or Scr by double-staining with Annexin V and PI (n = 5). H) Quantitative real-time PCR analysis of *ATF4* in WT NPCs infected with Ad-ATF4 or Control (n = 4). I) Quantitative real-time PCR analysis of *ZnT3* in WT NPCs infected with Ad-ATF4 or Control (n = 4). J-K) Representative FACS plots (J) and quantification (K) of the percentage of Zinquin^{+} cells in WT NPCs infected with Ad-ATF4 or Control (n = 6). L-N) Representative FACS plots (L) and quantifications of death cells (M, Annexin V^{+}/PI^{-}; N, Annexin V^{+}/PI^{+}) in WT NPCs infected with Ad-ATF4 or Control by double-staining with Annexin V and PI (n = 6). Data were presented as mean ± SEM. Significance was calculated by unpaired two-tailed Student's *t*-test. $^{*}p < 0.05$, $^{**}p < 0.01$, and $^{***}p < 0.001$ were considered to be significant for comparison of *WFS1*$^{-/-}$ Scr and *WFS1*$^{-/-}$ shATF4 or Control and ATF4 OE.

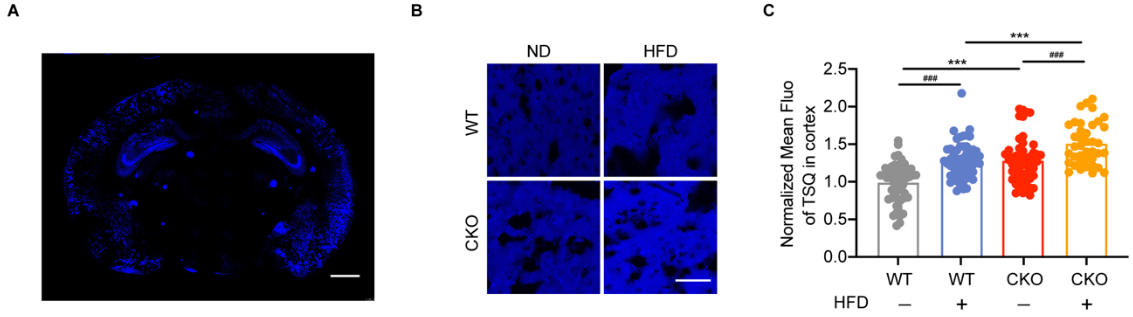

Figure. S8. Zinc levels are significantly elevated in HFD-induced obese mice

A) Representative image of TSQ fluorescence in the mouse brain. Scale bar, 1 mm. B) Representative images of TSQ fluorescence of the cerebral cortex in ND or HFD-fed WT and CKO mice. Scale bar, 50 µm. C) Quantification of the normalized intensity of TSQ in the cerebral cortex of ND or HFD-fed WT and CKO mice (WT + ND, n = 56; WT + HFD, n = 55; CKO + ND, n = 52; CKO + HFD, n = 39). Data were presented as mean ± SEM. Significance was calculated by unpaired two-tailed Student's *t*-test. $^{*}p < 0.05$, $^{**}p < 0.01$, and $^{***}p < 0.001$ were considered to be significant for the comparison of WT and CKO; $^{\#}p < 0.05$, $^{\#\#}p < 0.01$, and $^{\#\#\#}p < 0.001$ were considered to be significant for comparison of HFD treatment.

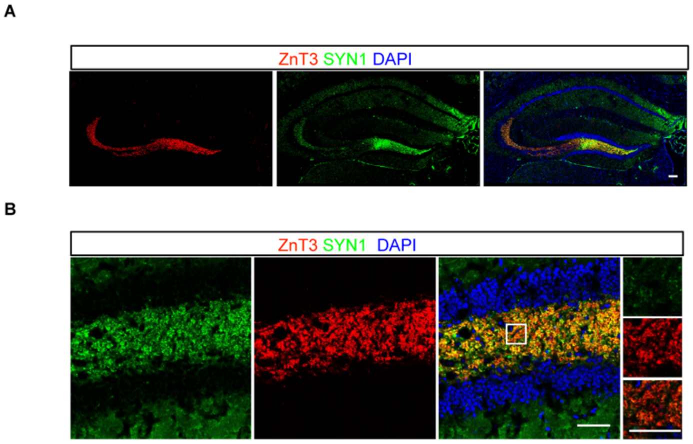

Figure. S9. The localization of ZnT3 on synaptic vesicle membranes within mossy fiber boutons in the hippocampus of mice

A) Immunostaining for ZnT3 (Red), SYN1 (Green), and DAPI (Blue) in the hippocampus of WT mice. Scale bar, 100 µm. B) Immunostaining for ZnT3 (Red), SYN1 (Green), and DAPI (Blue) in the hippocampus of WT mice. Scale bar, 50 µm.

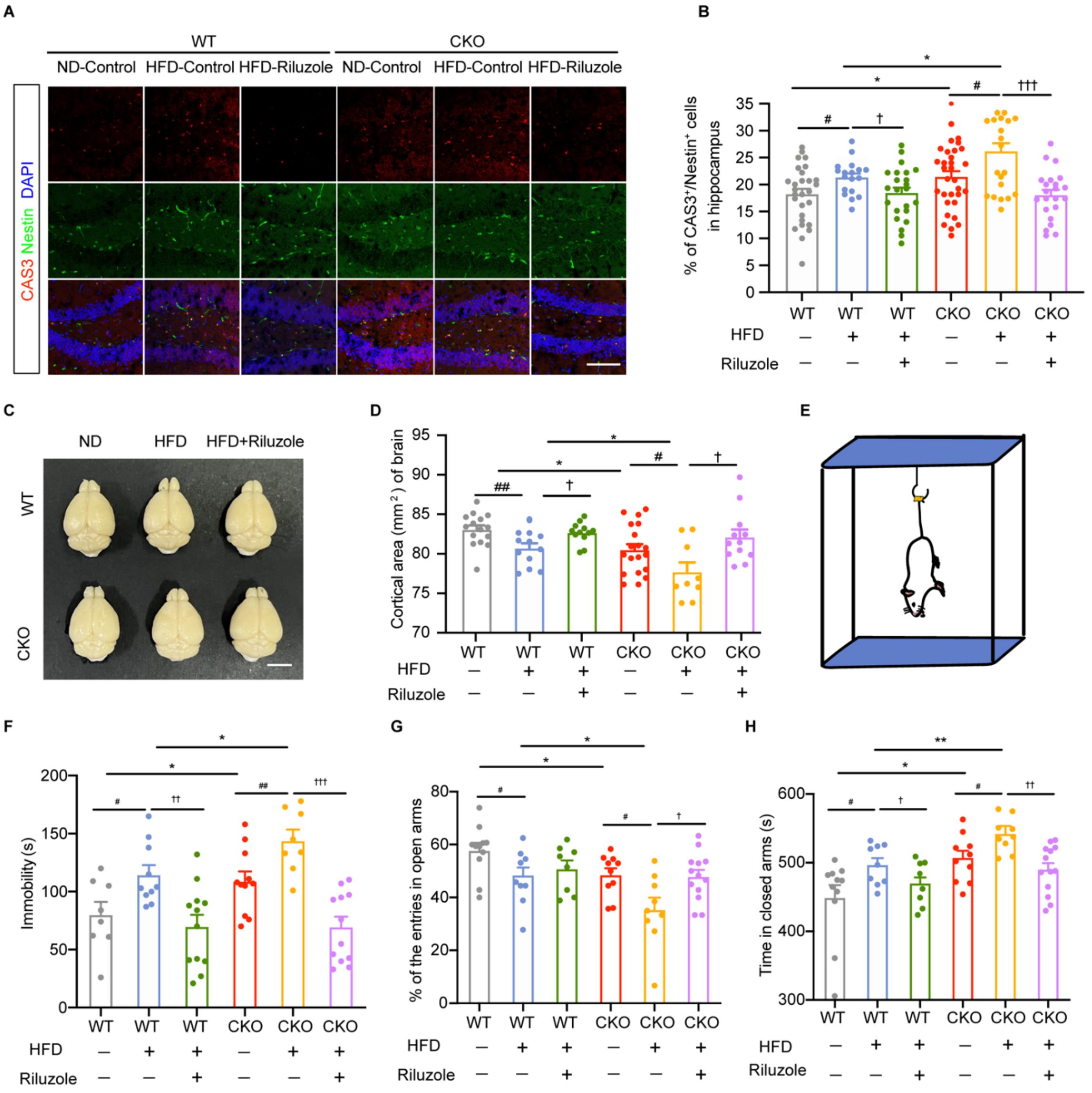

Figure. S10. Riluzole rescues HFD-induced smaller brain size and depression

A) Immunostaining for CAS3 (Red), Nestin (Green) and DAPI (Blue) in the hippocampus of ND or HFD-fed WT and CKO mice with riluzole or vehicle. Scale bar, 50 μm. B) Quantification of the percentage of $CAS3^+$ cells among the total number of $Nestin^+$ cells in the hippocampus of ND or HFD-fed WT and CKO mice with riluzole or vehicle (WT + ND + Control, n = 28; WT + HFD + Control, n = 18; WT + HFD + riluzole, n = 23; CKO + ND + Control, n = 32; CKO + HFD + Control, n = 22; CKO + HFD + riluzole, n = 22). C) Representative bright-field images of the brain in ND or HFD-fed WT and CKO mice treated with riluzole or vehicle. Scale bar, 5 mm. D) Quantification of the cortical area (mm^2) of the

brain in ND or HFD-fed WT and CKO mice treated with riluzole or vehicle (WT + ND, n = 15; WT + HFD + Control, n = 12; WT + HFD + riluzole, n = 12; CKO + ND, n = 19; CKO + HFD + Control, n = 9; CKO + HFD + riluzole, n = 12). E) Schematic of tail suspension test. F) Analysis of immobility time in ND or HFD-fed WT and CKO mice treated with Riluzole or vehicle in tail suspension test (WT + ND, n = 8; WT + HFD + Control, n = 10; WT + HFD + riluzole, n = 12; CKO + ND, n = 11; CKO + HFD + Control, n = 8; CKO + HFD + riluzole, n = 12). G-H) Analysis of the number of entries in open arms (J) and immobility time (K) in ND or HFD-fed WT and CKO mice treated with riluzole or vehicle in the elevated plus maze test (WT + ND, n = 11; WT + HFD + Control, n = 9; WT + HFD + riluzole, n = 8; CKO + ND, n = 11; CKO + HFD + Control, n = 9; CKO + HFD + riluzole, n = 13). Data were presented as mean ± SEM. Significance was calculated by unpaired two-tailed Student's *t*-test. $^{*}p < 0.05$, $^{**}p < 0.01$, and $^{***}p < 0.001$ were considered to be significant for the comparison of WT and CKO; $^{\#}p < 0.05$, $^{\#\#}p < 0.01$, and $^{\#\#\#}p < 0.001$ were considered to be significant for comparison of HFD treatment; $^{\dagger}p < 0.05$, $^{\dagger\dagger}p < 0.01$, and $^{\dagger\dagger\dagger}p < 0.001$ were considered to be significant for comparison of riluzole treatment.

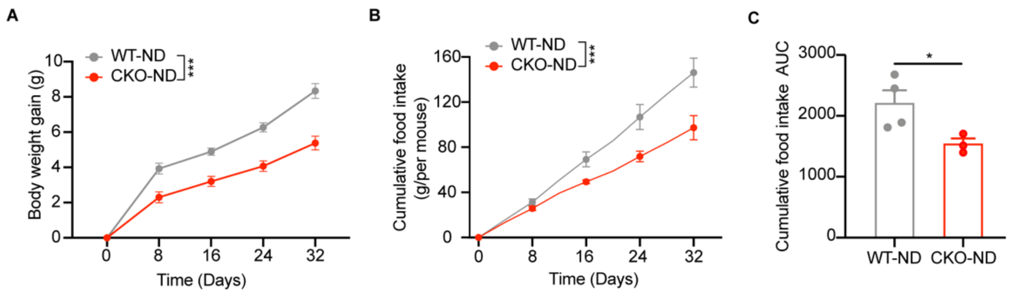

Figure. S11. ***WFS1* deficiency affects the food intake in mice**

A) Body weight gain in WT and CKO mice for 32 days from 6 weeks (WT, n = 11, CKO, n = 9). B-C) Body weight gain and Cumulative food intake in WT and CKO mice for 32 days from 6 weeks (WT, n = 4 group, CKO, n = 3 group). Data were presented as mean ± SEM. Significance was calculated by unpaired two-tailed Student's *t*-test. $^{*}p < 0.05$ was considered to be significant for comparison of WT and CKO.

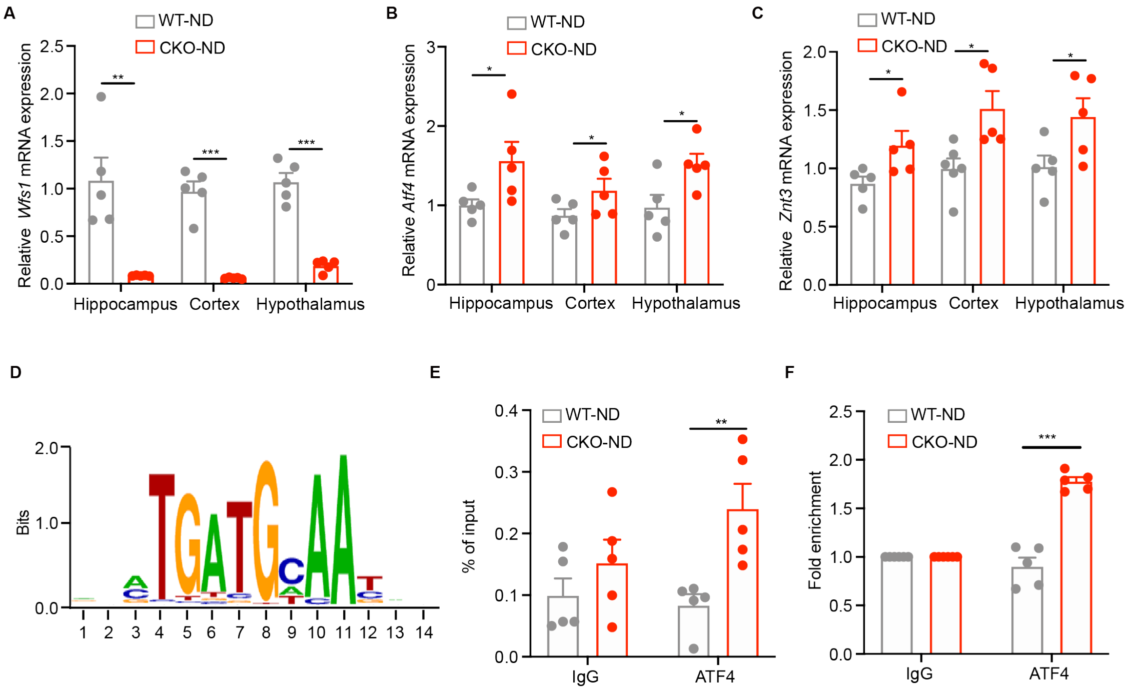

Figure. S12. The enhanced ATF4 binding to ZnT3 promoter in CKO mice

A-C) Quantitative real-time PCR analysis of *Wfs1*, *Atf4*, and *Znt3* in hippocampus, cortex, and hypothalamus region of WT and CKO mice (n = 5). D) *Znt3* promotor binding motifs of ATF4. E-F) ChIP analysis. % of Input (E) and relative fold change (F) of ATF4 enrichment at *Znt3* promotor in the cortex region of WT-ND and CKO-ND mice (n = 5). Data were presented as mean ± SEM. Significance was calculated by unpaired two-tailed Student's *t*-test. $^{*}p < 0.05$, $^{**}p < 0.01$, and $^{***}p < 0.001$ were considered to be significant for the comparison of WT and CKO.

Table S1. List of primer sequences used for qPCR in humans

Gene	Forward (5' - 3')	Reverse (3' - 5')
GAPDH	GGAGCCAAACGGGTCATCATCTC	GAGGGGCCATCCACAGTCTTCT
WFS1	CTCAACAGCTGCACCGCTGT	CCATCGTGCTCGTTGACCTG
ZnT1	CTCGCGTTAAGAGCACCCG	CAATTTCAGCCCGTTGGAGTT
ZnT3	CACCCGCACCATGACCTTT	AAGGCCATTAACAGGTTGGCA
ZnT4	TGGAAGCGCCTCAAATCTATG	CAGTAAGGAATCATCGTCGGC
ZnT5	TCTATGGCGTGCTGACCAATA	GCATGGCTATGGCATGGTG
ZnT9	AGGGAGTATGGCTCAAAGTACA	GGGACTTCGTCGTCTGATTTTTC
ZIP3	GCACCCCAATGTGATCTGC	CGCTACACAAGTCGTAGTCTGG
ZIP6	ATGCAAGTCACCACCATAGTCA	ACGTGGAATCAAAATAGGCACT
ZIP9	CATGCTGGCTTAGAGCGGAAT	TGGTGCTGCCAATGCAAAGA
ZIP10	TTTCACTCACATAACCACCAGC	GTGATGACGTAGGCGGTGATT
ZIP14	AAGGCCCTACTCAACCACCT	CGACTGCTCGCTGAAATTGTG

Table S2. List of primer sequences used for qPCR in mouse

Gene	Forward (5' - 3')	Reverse (3' - 5')
Gapdh	GGAGCCAAACGGGTCATCATCTC	GAGGGGCCATCCACAGTCTTCT
Wfs1	CGGGAAGAAACGGACAGAGC	CGTAGGTAGTGTTTGCCCAC
Atf4	ATGGCGCTCTTCACGAAATC	ACTGGTCGAAGGGGTCATCAA
Znt3	GTGCTGGGAGACCTCCTTC	ACGGGTTCAAATTCCACACTC

Table S3. List of primer sequences used for ChIP-PCR in humans

Gene	Forward (5' - 3')	Reverse (3' - 5')
ZnT3	CCAATTTTAAGGAGGAAACA	TTACTCCAGGTAATAGGAG

Table S4. List of primer sequences used for ChIP-PCR in mouse

Gene	Forward (5' - 3')	Reverse (3' - 5')
Znt3	CAGGAAGCAACTGAGGCAAGAAG	ACCCCTCCTTCAGTGTGACTTGA
